# Functionalization of vertically aligned carbon nanotubes

**DOI:** 10.3762/bjnano.4.14

**Published:** 2013-02-22

**Authors:** Eloise Van Hooijdonk, Carla Bittencourt, Rony Snyders, Jean-François Colomer

**Affiliations:** 1 Research center in Physics of Matter and Radiation, University of Namur, Namur, Belgium; 2 Chimie des Interactions Plasma-Surface, Research Institute for Materials Science and Engineering, University of Mons, Mons, Belgium,; 3 Materia Nova Research Center, Mons, Belgium

**Keywords:** aligned, carbon nanotubes, fluorination, functionalization, graphene, nitration, oxidation

## Abstract

This review focuses and summarizes recent studies on the functionalization of carbon nanotubes oriented perpendicularly to their substrate, so-called vertically aligned carbon nanotubes (VA-CNTs). The intrinsic properties of individual nanotubes make the VA-CNTs ideal candidates for integration in a wide range of devices, and many potential applications have been envisaged. These applications can benefit from the unidirectional alignment of the nanotubes, the large surface area, the high carbon purity, the outstanding electrical conductivity, and the uniformly long length. However, practical uses of VA-CNTs are limited by their surface characteristics, which must be often modified in order to meet the specificity of each particular application. The proposed approaches are based on the chemical modifications of the surface by functionalization (grafting of functional chemical groups, decoration with metal particles or wrapping of polymers) to bring new properties or to improve the interactions between the VA-CNTs and their environment while maintaining the alignment of CNTs.

## Introduction

Carbon nanotubes (CNTs) have stirred the curiosity of the scientific community for two decades now. They consist of layers of graphene rolled up on themselves in order to form cylinders often closed at the two ends by fullerenic caps. Either they are encased one in another in a coaxial way and are called multiwalled carbon nanotubes (MWCNTs) [1], or they consist of a single layer and are categorized as single-walled carbon nanotubes (SWCNTs) [2]. SWCNTs are generally assembled in two-dimensional, compact, wide ropes called bundles [3]. The structure of the nanotubes determines the majority of their properties. Their symmetry is related to the orientation of the hexagonal lattice with respect to the axis of the tube (chirality). Depending on the chirality, a carbon nanotube shows either metallic or semiconductor behavior [4]. Thus, this allotrope of carbon exhibits exceptional morphological, physical and chemical properties: high aspect ratio (a length-to-diameter ratio greater than 10 000 and as high as 132 000 000) [5], an extremely high conductance [6], a high structural flexibility [7], and a high thermal conductance [8]. With a high tensile strength [9] and elastic modulus [10], CNTs form the strongest and stiffest material that humans have created. These properties offer a wide range of potential applications [11–12], for electronic devices, energy storage and transport, nanocomposite materials, and nanomedicine.

The as-synthesized product is macroscopically seen as a black powder whereas microscopically the CNTs are randomly oriented in an entangled spaghetti-like configuration. Until recently, practical applications of CNTs had been limited by issues related to their synthesis. The first important research outcomes in this area were the synthesis on a large scale, the reproducibility, and the control of the diameter and number of walls of the CNTs using different synthesis techniques such as arc discharge, laser ablation or chemical vapor deposition. After these issues had been addressed, the focus in carbon nanotube research shifted towards obtaining control in the engineering of organized architectures with determined orientations, such as vertically aligned carbon nanotubes (VA-CNTs). Because of the strong anisotropy of the CNT properties, the orientation of the longitudinal direction of the CNTs is often requested in many applications such as field-emission displays, chemical or biological sensors, or polymer fillers. The advantages of using VA-CNTs include an excellent alignment of the nanotubes, a good electrical and thermal conductivity, and uniform length. Nevertheless, a key challenge to be overcome for achieving actual applications is the tuning of the CNT surface properties. In this context, functionalization (i.e., the grafting of chemical groups (molecules or particles) on the surface of the nanomaterial) has been reported to give excellent results, with the drawback that it negatively impacts on the alignment of the VA-CNT. To date, different functionalization methods have been reported [13], all being well-known and controlled for non-aligned CNTs (single or multiwalled). These methods can be divided into two major functionalization strategies. The first is “endohedral functionalization”, in which the CNT functionalization is obtained by filling the inner cavity with guest nanoparticles [14] or molecules [15]. This can be achieved by using colloidal suspensions or applying special thermal or chemical conditions (called “wet chemistry”). In the second strategy, termed “exohedral functionalization”, only the external sidewall of the CNTs is functionalized. During the functionalization, functional groups or nanoparticles can form a covalent (chemical) or a non-covalent (physical) bond with the CNT surface. The covalent functionalization (creation of a chemical bond between the CNT and the functional group or nanoparticle) can occur at the fullerenic caps, which are more reactive than the CNT sidewalls [16], at the defects, or exclusively at the sidewalls of the nanotubes. The non-covalent functionalization (creation of a physical bond between the CNT and the chemical group or particle) involves for instance CNTs wrapped by polymers. Several methods involving the functionalization of the non-aligned CNTs, such as wet-chemistry, are not applicable to VA-CNTs if the alignment of the nanotubes must be preserved. A review devoted to the functionalization of non-aligned CNTs was written by Balasubramanian and Burghard in 2005 [17]. Regarding the VA-CNTs, the functionalization method should be well-controlled, restricting damage to the nanotubes and their arrangement [18–20]. Another, important characteristic of a post-growth treatment is the removal of the amorphous carbon layers that can be often observed on the as-grown CNTs [21]. In this context, physical functionalization, such as plasma treatment where the functionalization features depend on the plasma parameters, has been reported. This technique allows an optimal tunable chemical modification of the CNT surface. A detailed review devoted to the surface treatment of non-aligned carbon nanotubes by different plasma technologies was published by Ruelle et al. [22] in 2011.

The present review is focused on the different methods available to functionalize vertically aligned carbon nanotubes, including plasma functionalization, wet chemical functionalization, and also dry gas-phase functionalization. Before presenting these methods, we briefly discuss different ways to synthesize vertically aligned carbon nanotubes and the existing approaches to obtain patterning of vertically aligned carbon nanotubes. A part of this review is also dedicated to a frequent consequence of the functionalization (voluntarily searched or not): the bundling of CNTs.

## Review

### 1 Synthesis of vertically aligned CNT arrays

**Figure 1:**
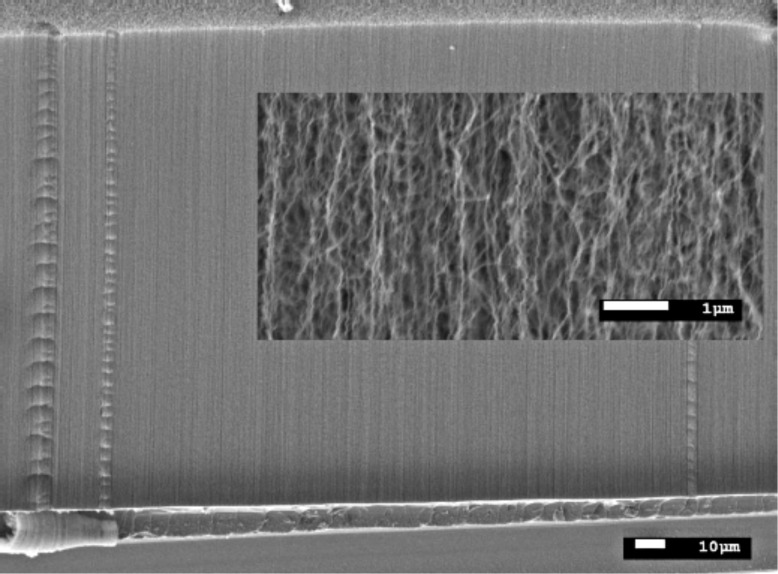
Example of scanning electron microscopy image of carbon nanotubes aligned perpendicularly to the substrates and synthesized on 30 Å Fe/300 Å Al_2_O_3_/SiO_2_/Si for 30 min (C_2_H_4_/H_2_ 35/130 sccm) at 750 °C.

Considerable progress has been made in the synthesis of vertically aligned CNTs since the first report, in 1996, by Li et al. [23] showing the synthesis of aligned CNTs from acetylene chemical vapor deposition (CVD) catalyzed by iron nanoparticles embedded in mesoporous silica. In that work, the growth direction of not very straight, entangled-CNTs was related to the direction of the pores in the silica substrate, being perpendicular to the substrate surface if the pores were vertical. Later, in 1998, the synthesis of very straight, aligned multiwalled CNTs on nickel-coated glass at temperatures as low as 666 °C was obtained by Ren et al. [24] using plasma-enhanced hot-filament chemical vapor deposition (PE-HF-CVD). Reported in 1999 by Fan et al. [25], a key achievement was the engineering of vertically oriented CNT-arrays by using CVD of ethylene, size-controlled Fe catalytic particles, and nanotube positioning by substrate patterning. The mechanism of the alignment of the CNTs was proposed to be due to the van der Waals forces where the outer wall of each nanotube interacts with the outer walls of neighboring nanotubes to form large bundles with sufficient rigidity to keep the growth direction. The density of nanotubes promotes the aligned growth (Figure 1). More recently, Hata et al. (2004) reported the growth of superdense, vertically aligned single-wall carbon nanotubes with heights up to several millimeters achieved by increasing the activity and lifetime of the Fe-based catalysts during the ethylene CVD by adding small amounts of water vapor, which acts as an oxidizer [26]. And, in 2006, Yamada et al. reported the selective (85%) growth of double-walled carbon nanotubes also by water-assisted CVD [27]. The development of CNT-based applications requires an easy, secure, inexpensive way of industrialization producing large VA-CNTs surfaces with, obviously, high-quality and well-controlled properties (homogeneity, length, doping, etc.). A great progress in this context has been recently realized by the IRAMIS/SPAM/Francis Perrin laboratory (France) [28]. Using the aerosol-assisted CVD technique, they synthesized VA-CNTs carpets over large surfaces such as a 30 cm in diameter silicon wafer.

**Figure 2:**
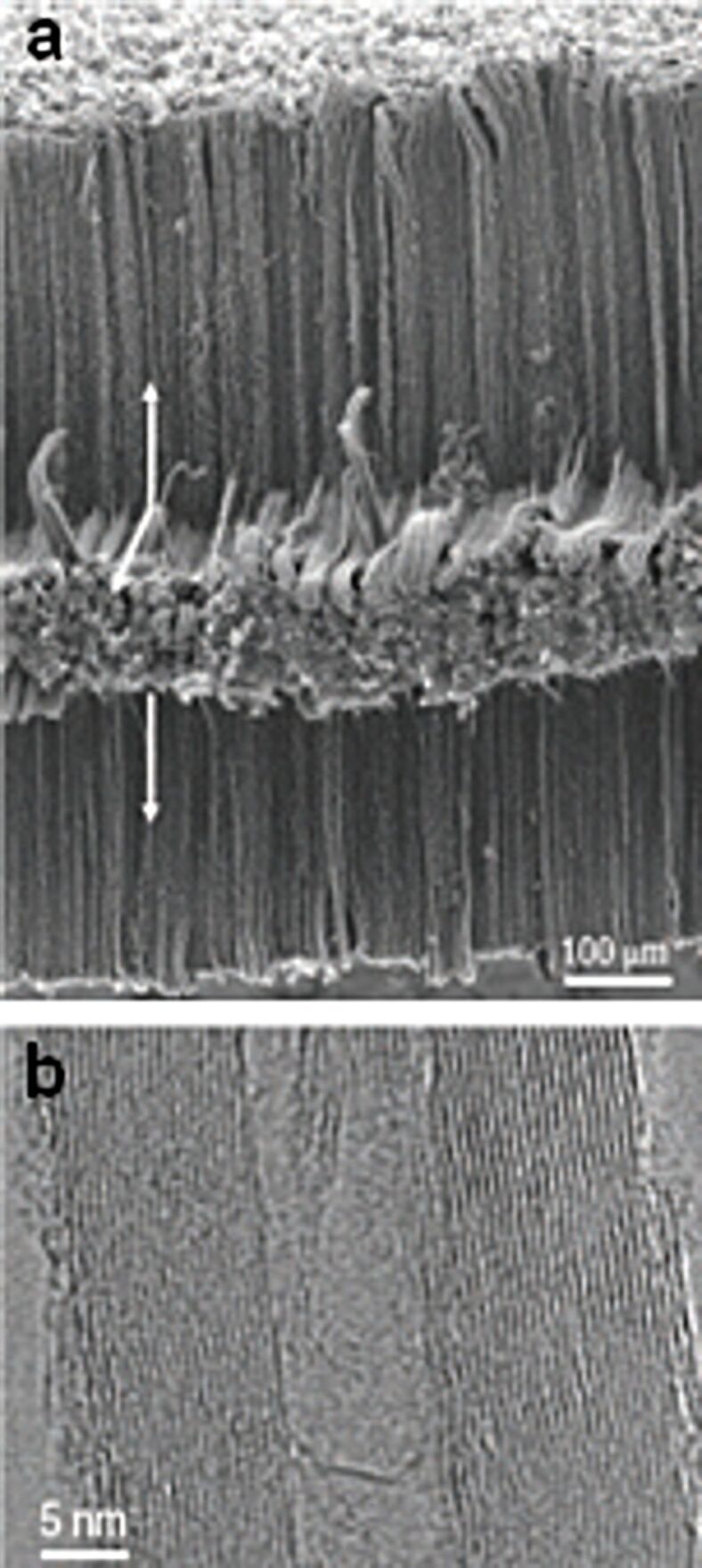
(a) SEM and HR-TEM images of aligned MWCNTs grown in three dimensions on Inconel substrates by floating catalyst CVD. The directions of CNT growth are indicated by arrows. (b) High-resolution transmission electron microscopy image of a typical nanotube grown in this way, showing the well-graphitized walls of the MWNT. Adapted with permission from [33]. Copyright 2006 Nature Publishing Group.

Among the different methods used to synthesize non-aligned CNTs, such as chemical vapor deposition (CVD), electrical arc discharge, or laser ablation, CVD has been reported to be the best for the synthesis of VA-CNTs. Different versions of CVD for the synthesis of VA-CNTs have been used, which can be mainly divided in two types: those involving a single synthesis step and those requiring double step synthesis. The single-step synthesis is based on the pyrolysis of organometallic precursors such as metallocenes [29–30]; this method was called “floating catalyst CVD” because it does not require the preparation of catalyst particles. Floating catalyst CVD involves vaporization and sublimation of a catalyst precursor on a substrate that is then introduced in a high-temperature zone where the growth of the CNTs takes place. Whether the CNT growth starts during the floating stage [31], or from metal particles deposited on substrates or on the walls of the reactor leading into the furnace, followed by the tip-base growth mechanism [32] has been the subject of discussion. This technique allows the preparation of well-aligned and three-dimensional architectures of CNTs on conductive as well as nonconductive substrates (Figure 2) [33].

In the double-step synthesis, firstly catalytic nanoparticles with controlled size and distribution are prepared, and then the growth of CNTs is performed. The nanoparticles can be obtained by physical methods (for example, Physical Vapor Deposition (PVD)) or chemical methods by using precursor solutions for the catalysts, which are then deposited by dip or spin coating or, alternatively, are filled into nanoporous architectures serving as templates for the CNT growth.

Plasma vapor deposition is an efficient method for the preparation of thin films of metal catalysts with well-defined thicknesses. The catalytic nanoparticles are formed by annealing the film; the size and distribution of the nanoparticles are controlled by the thickness of the deposited film, the annealing parameters, and the type of the substrate (and added buffer layers) used for depositing the films [34–35]. In the case of chemical methods for catalyst particle production, the chemistry of the catalyst solution is crucial for determining the size of the nanoparticles, whereas their distribution is mainly determined by the deposition technique, for example spin [36] or dip [37] coating. The most common solutions consist of metal salts diluted in alcoholic solutions; however, stable solutions by using surfactants [38] or applying a sol–gel process [39] can be also used. The main disadvantage of the chemical method is the difficulty of optimizing the catalyst solution. The template-based approach in which the catalysts are deposited within the pores of nanoporous templates (for example, anodic aluminum oxide (AAO) membranes) allows design of a wide range of nanostructures with particular geometries, including aligned and monodispersed CNTs [40].

After the catalyst preparation, the next step is the synthesis of the VA-CNTs. This can be performed by thermal chemical vapor deposition (CVD) or plasma enhanced chemical vapor deposition (PECVD). The PECVD includes different plasma techniques, such as DC plasma [41], radio-frequency plasma [42], or microwave plasma [43]. The main difference is that the plasma in the PECVD provides a highly reactive environment compared with thermal CVD, allowing lower synthesis temperatures. An activation energy of about 1.2–1.8 eV [44–45] characterizes the thermal CVD while a lower activation energy of ≈0.3 eV [46] was reported for the PECVD. These energies are defined taking into account the four fundamental steps occurring during the CVD growth of CNTs: adsorption of the gas precursor molecule on the catalyst surface, dissociation of the precursor molecule, diffusion of the growth species in/on the catalyst particle, and nucleation and incorporation of carbon into the growing structure. Hoffman et al. [47] demonstrated that the limiting step in the determination of the activation energy for thermal CVD is the dissociation of the precursor molecule and, for PECVD, is the carbon diffusion on the catalyst. Thanks to the low temperatures in PECVD compared to the temperatures in thermal CVD, substrates that could be damaged at high temperature (for example glass) can be used as a support for VA-CNTs or their synthesis can be performed directly integrated in devices [48]. The CVD involves the decomposition of hydrocarbon gas molecules on the surfaces of catalyst nanoparticles, followed by carbon diffusion through the nanoparticles resulting in carbon precipitation at the surface and the nanotube growth. Two growth mechanisms were proposed: tip or base related growth [46]. In the first case, the catalyst nanoparticle comes off the substrate and, after synthesis, is observed at the top of the CNT. In the second case, the particle remains attached to the substrate. The common explanation for this difference is based on the adhesion force between the catalyst and the substrate. It is reported that a strong (weak) interaction furthers the base (tip) related mode. However, Gohier et al. [49] demonstrated that, during the catalytic chemical vapor deposition growth process, the particle size also plays a key role in the determination of the growth mechanism. For a given substrate/catalyst pairing, tip-related growth is favored when the catalyst particles are large (>>5 nm), giving multiwalled CNTs (>10 walls); while base-related growth is promoted for small particles (<5 nm), producing single or few-wall CNTs (typically less than seven walls). Gauthier et al. explained the shift between both growth modes using two different pathways for carbon diffusion [49].

**Figure 3:**
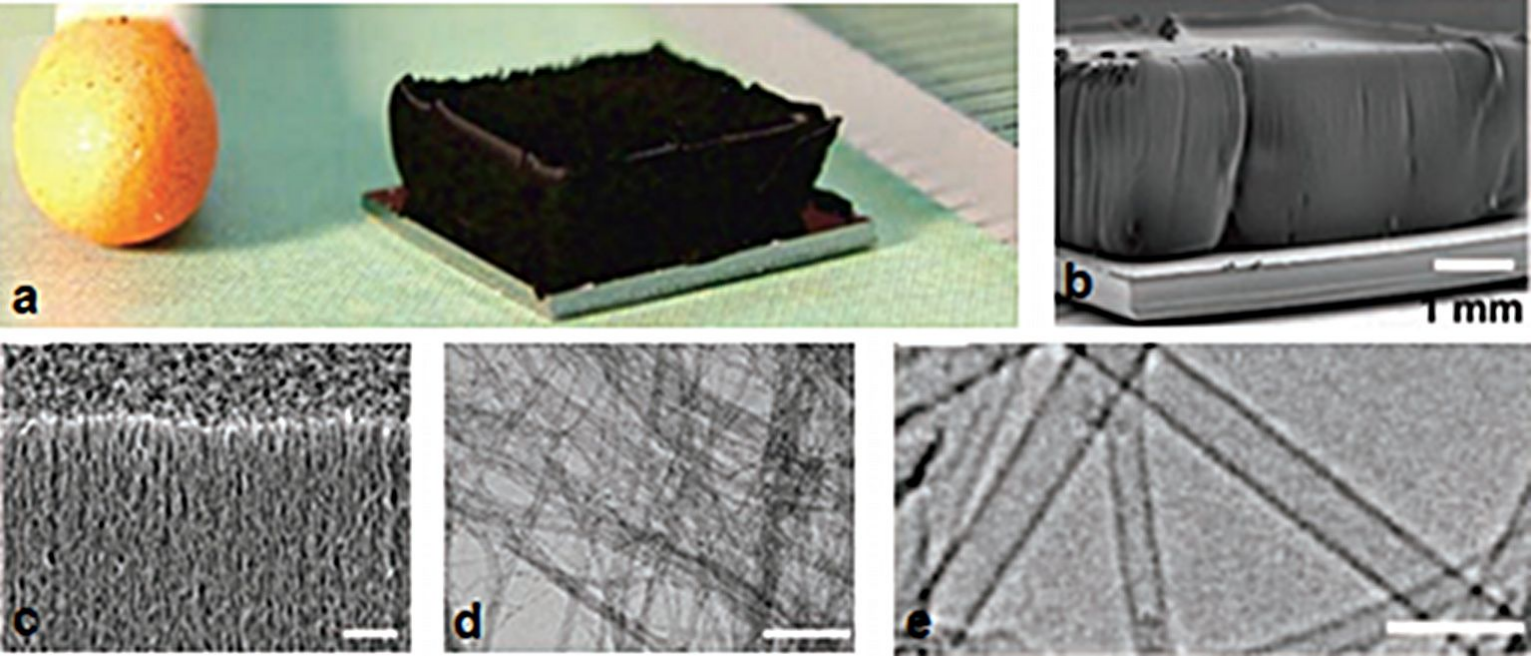
SWCNT forest grown with water-assisted CVD. (a) Picture of a 2.5 mm tall SWCNT forest on a 7 mm by 7 mm silicon wafer. A matchstick on the left and ruler with millimeter markings on the right is for size reference. SEM images of the same SWCNT forest with scale bars of 1 mm (b) and 1 μm (c), respectively. TEM images of the nanotubes with scale bars of 100 nm (d) and 5 nm (e), respectively. Adapted with permission from [26]. Copyright 2004 American Association for the Advancement of Science.

The main constraint for the growth of CNTs is the poisoning of the catalyst due to encapsulation by amorphous carbon. In 2004, Hata et al. [26] reported the growth of VA-CNTs with millimeter length (Figure 3). By adding a small amount of an oxidizer during the CVD synthesis the poisoning of catalyst nanoparticles is prevented, extending the catalyst lifetime [50]. Another positive effect of the use of the oxidant is the inhibition of Ostwald ripening if injected during the annealing treatment of the catalyst film; the Ostwald ripening causes the formation of large particles thus reducing the yield of the CNT synthesis [51]. The usual oxidizer agent employed is water vapor, but in general oxygen-containing compounds such as alcohols or ethers are efficient. Alternatively the synthesis of VA-CNTs can be performed by using an alcohol as a carbon source with the advantages that the alcohol serves also as a weak oxidizer [52]. The two leading methods currently used to synthesize vertically aligned carbon nanotubes are the ferrocene-catalyzed growth of aligned multiwalled carbon nanotubes reported by Talapatra et al. [33] and the (super)-growth of ultrahigh-aligned single- (double-)walled carbon nanotubes on the catalytic system composed of thin Al_2_O_3_/Fe metal layers proposed by K. Hata, and co-workers [26–27].

In 2012, Zhu et al. [53] reported the synthesis of a hybrid material formed by VA-SWCNTs covalently bonded to graphene. The synthesis strategy is as follows. A sandwich consisting of a graphene substrate/catalyst layer/alumina layer is used. During the CNT growth, the catalyst and alumina layers are lifted up and the CNTs grow vertically aligned, directly on the graphene layer. As a result of this strategy, no seam exists between the graphene and the nanotubes carpet. The introduction of the top alumina floating buffer layer is a crucial step, it allows the control of the CNTs diameter while forming a covalent bond between both elements. The particularity of this hybrid material is that it extends to three dimensions the excellent electrical conductivities and the large specific surface areas (SSA) of SWCNTs and graphene. The covalent bond between both elements leads to an ohmic contact at the junction. In parallel to this efficient electrical contact, a SSA between 2,000 and 2,600 m^2^·g^−1^ was measured. This work opens perspectives for potential applications in energy storage and nanoelectronic devices.

Outstanding progress in the synthesis of aligned carbon nanotubes has been reported; several studies show promising results in the control of the density, the length, the diameter, number of walls, and alignment of the vertically aligned nanotubes. We intended to give a brief overview of the synthesis techniques and the main achievements in this field. For further reading, detailed literature can be found in [54].

### 2 Patterning of VA-CNT arrays

For optimal engineering in applications such as field emitters or sensors, the location of the VA-CNTs and the design of their growth area are of primary importance. Two main strategies have been used to pattern VA-CNTs: the first is pre-patterning of the catalysts/substrates in order to form islands with desired size and shape and, thus, to pattern the growing VA-CNTs; the second is to directly pattern the VA-CNTs arrays by selective etching.

Most of the reported strategies are related to the first method in which the pre-patterning of the substrate is obtained by using shadow masks, photolithography and electron-beam-lithography (e-beam lithography). The first example, was given by Fan et al. [25]. These authors synthesized aligned carbon nanotubes in tower-like arrays by patterning the substrate with Fe films using electron beam evaporation through shadow masks, patterned with squared openings of well-defined size. The shape and size of the holes in the shadow masks determine the resulting shape of the patterned aligned nanotubes. For example, columns of aligned nanotubes can be obtained by using substrates patterned with catalyst dots. High lateral resolution in the patterning of the substrate for site-selective growth of VA-CNTs can be achieved by photolithography with the use of high-contrast films as photomasks with features on the microscale [55–56]. The use of other lithographic techniques such as soft-lithographic approach [57] or e-beam lithography [58], to pattern VA-CNTs, have been reported.

The soft-lithographic approach consists of micro- or nanopatterning processes mainly using two methods applied for aligned carbon nanotubes: microcontact printing and solvent-assisted micromolding techniques. Microcontact printing involves the use of a poly(dimethylsiloxane) (PDMS) stamp with self-assembled monolayer (SAM) coating to print the substrate surface by transfer of the SAM (e.g., alkysiloxane). A photoresist solution is deposited in the areas not covered by the SAM, patterning the resulting polymer [57]. After the removal of SAM and the carbonization step of the PDMS, aligned carbon nanotubes can grow in the areas from which the SAM was removed. This technique has also been use to print catalysts, instead of SAMs, to synthesize free-standing SWCNTs on regularly patterned silicon with tower-like structures [59] or aligned multiwalled carbon nanotubes [60]. The second method, solvent-assisted micromolding consists of printing a drop of photoresist solution by pressing with a PDMS stamp with a patterned relief structure on its surface. After drying, a patterned polymer is obtained and, consequently, after the carbonization step, patterned aligned carbon nanotubes can grow [57,61]. The disadvantage of the use of polymer in the lithography techniques described previously is the carbonization step, that is necessary to transform the polymer into carbon black areas, which then remains on the surface of the substrate to impede the growth of aligned nanotubes in these regions. To overcome this problem, plasma patterning can be employed to polymerize a resin in a patterned way. The obtained plasma-patterned substrate is then subjected to a pyrolysis of iron(II) phthalocyanine (FePc) under Ar/H_2_ atmosphere at 800–1100 °C, which leads to the growth of vertically aligned carbon nanotubes in the plasma-patterned polymer-free regions. The highly cross-linked structure of the plasma-patterned polymer films guarantees the integrity of the polymer layer, remaining stable at the high temperatures involved in the CNTs growth process from FePc. The carbonization step, i.e., the inconvenient stage when using polymers in lithography techniques, can therefore be skipped with this method [62].

**Figure 4:**
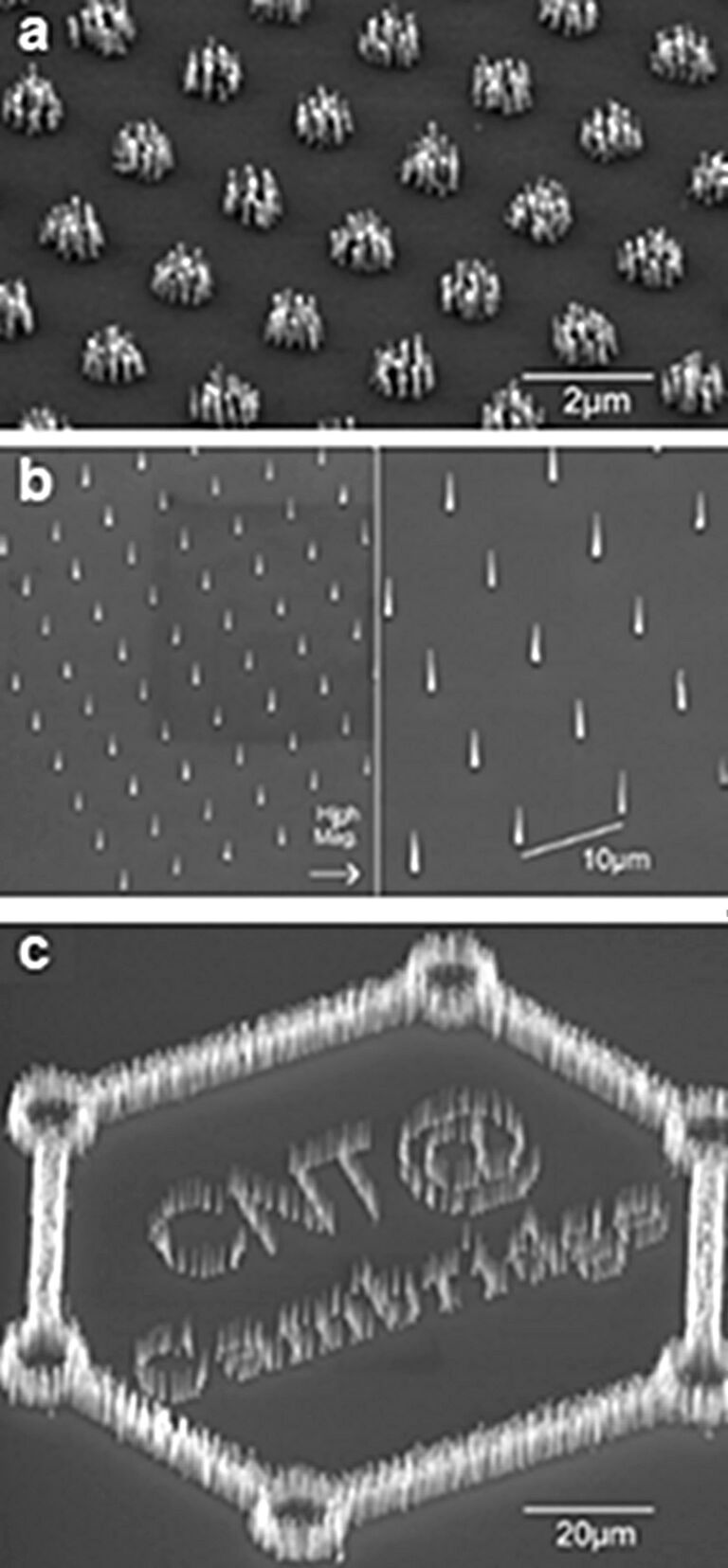
Various examples of nanotube arrays grown on Ni dots. (a) Bunches of nanotubes (about 100 nm in diameter) are deposited on 1 µm nickel dots, because the nickel catalyst film breaks up into nanoparticles. (b) Single nanotubes will grow when the size of the Ni dot is reduced to 100 nm, as only a single nanoparticle is formed from such a dot. (c) Demonstration of high yield, uniform, and selective growth of nanotubes at different densities. Adapted with permission from [63]. Copyright 2001 American Institute of Physics.

**Figure 5:**
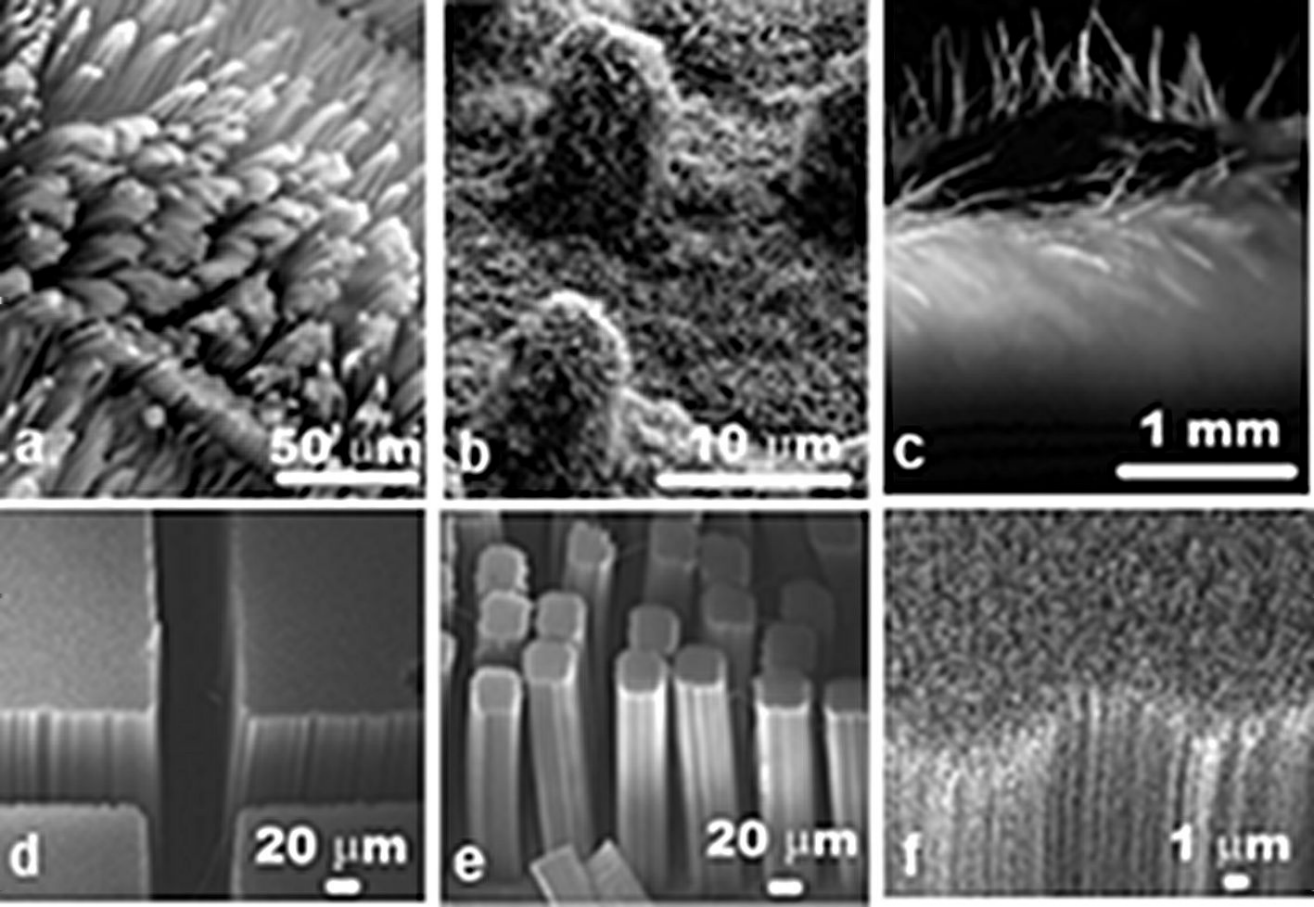
SEM images of natural (a) gecko setae, (b) a lotus leaf with hierarchical roughness, and (c) the hairy structure of lady’s mantle leaf. SEM images of synthetic setae made of micropatterned aligned carbon nanotubes where they act as spatulas (d-f). Adapted with permission from [66]. Copyright 2008 American Chemical Society.

An alternative technique used to pattern aligned carbon nanotubes is e-beam lithography. It allows the selective growth of single free-standing aligned multiwalled carbon nanotubes [58]. For that purpose, the catalyst film is deposited precisely in selected regions; the density of catalytic nanoparticles in the region can be controlled, with a remarkable demonstration of writing, using patterned aligned nanotubes (Figure 4) [63]. An interesting example of patterned aligned carbon nanotubes is the engineering of gecko-foot-mimetic dry adhesives. Patterning is employed to effectively reproduce the setae of gecko composed of many lobes, aimed at obtaining the remarkable self-cleaning abilities and the same properties, i.e., high shear and peeling forces (Figure 5) [64–66].

A further step in patterning VA-CNTs is the engineering of 3D-architectures. The formation of 3D-aligned carbon nanotube patterns is obtained by growing VA-MWCNTs with different lengths and packing densities on specific regions, with covered and uncovered photoresist films using a photolithographic approach. The surface areas covered and uncovered by the film lead to a difference in the aligned CNTs growth on these two regions and consequently to the formation of 3D aligned carbon nanotube patterns [67].

**Figure 6:**
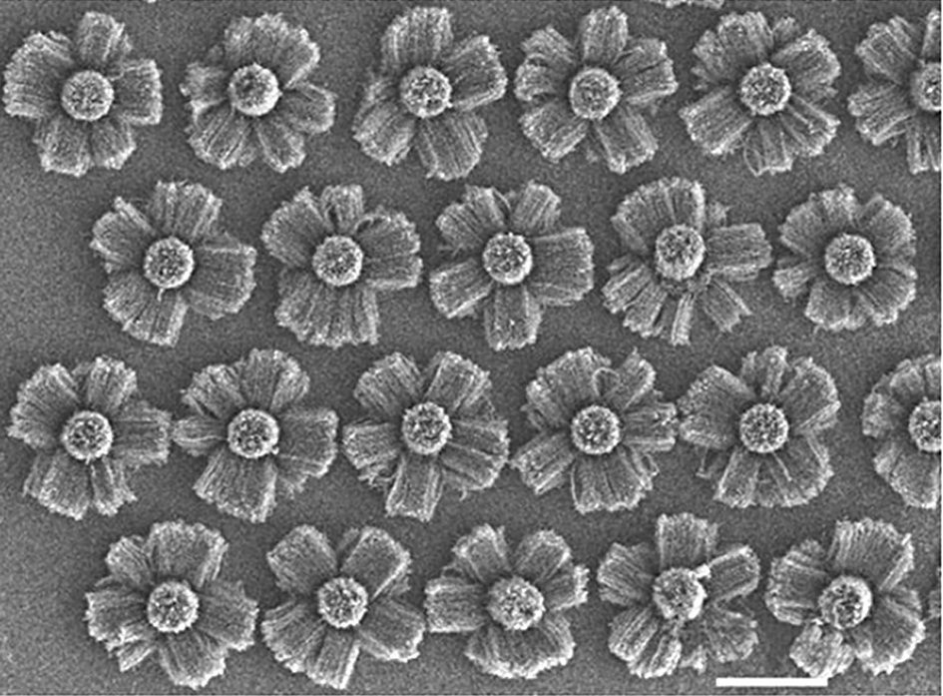
SEM image of beautiful repeating patterns of multiply oriented, organized nanotube structures on deep (about 5 μm) silica features (circular cross section), machined on silicon substrates. Growth in the vertical direction occurs from the top silica surface (seen as arrays emanating from the center of each pattern); growth on the sides occurs as horizontal arrays (sideways growth seen on each pattern) (scale bar, 50 µm). Adapted with permission from [69]. Copyright 2002 Nature Publishing Group.

Photolithography followed by dry and/or wet etching can be used to pattern silicon oxide in different shapes and thickness allowing the design of a wide range of organized nanotube structures. An example is the beautiful patterns of multiply oriented, organized, flower-like structures of nanotubes (Figure 6) [68–69]. A different method is the so-called “contact transfer” for producing micropatterns of the aligned carbon nanotubes by pressing on the adhesive layer of Scotch tape pre-patterned with a non-adhesive layer, followed by peeling the Scotch tape off to obtain positive and negative patterns [70]. 3D multicomponent micropatterns were synthesized by direct growth using masks produced by contact transfer [71]. Another example is the patterned growth of 3D interposed VA-SWCNTs and VA-MWCNTs by activating selective regions of iron substrates used for the synthesis of VA-MWCNTs with a thin layer of Al for the growth of VA-SWCNTs [72].

**Figure 7:**
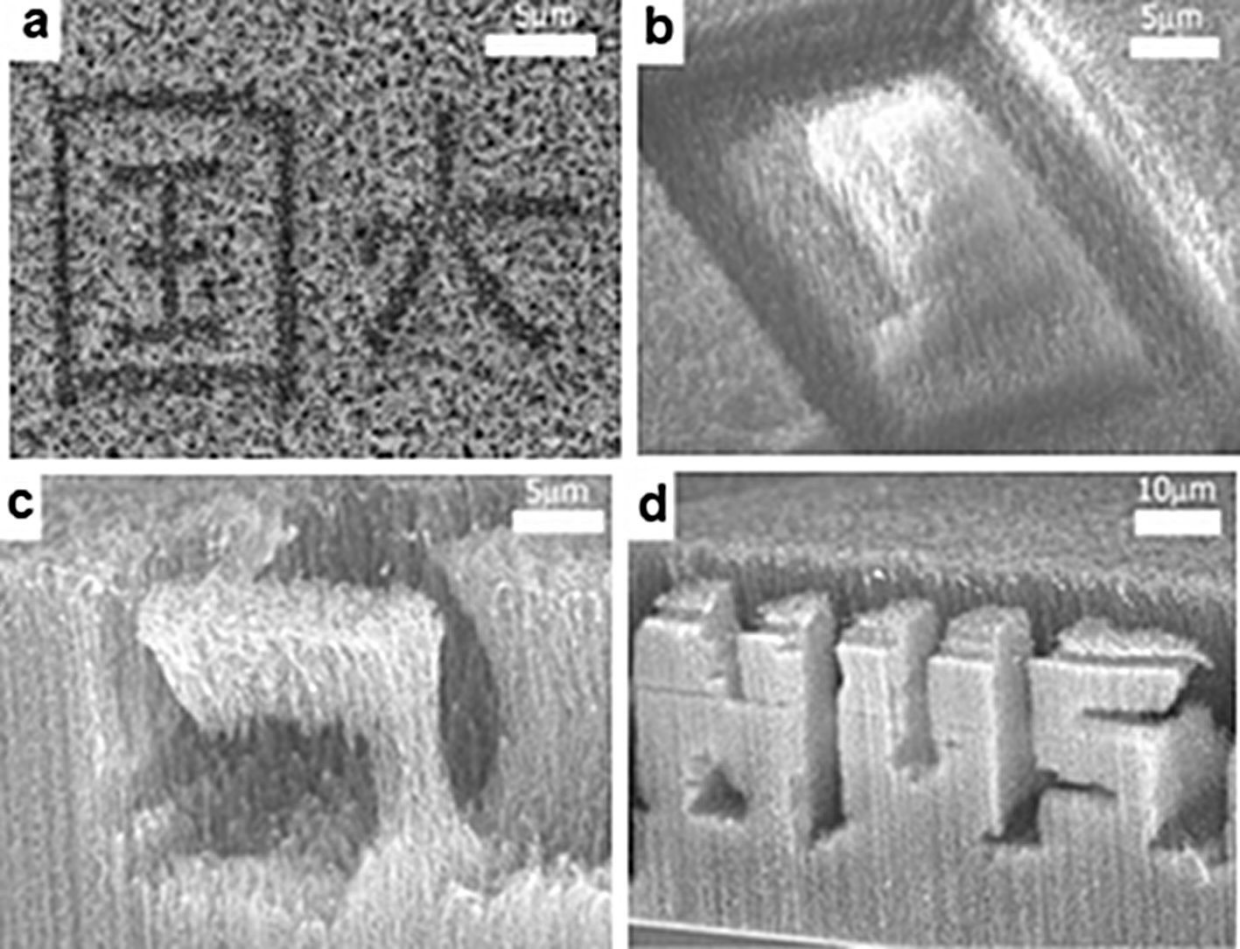
SEM images of (a) Chinese characters cut in 2D on a mat of CNTs, (b) a pyramidal structure cut by focusing the laser to different depths and scanning over the predetermined areas, (c) an inverted “L”, and (d) 3D letters “NUS” produced in a two-step process: cutting first from the side and then with the sample in face-up configuration. Adapted with permission from [73]. Copyright 2003 John Wiley and Sons.

Aligned carbon nanotubes can be directly etched by laser irradiation. A pruning method using a focused laser beam can be used to fabricate different 3D architectures. The spread of the beam causes the removal of a thin layer of aligned CNTs, and movable flaps of CNTs can be created by undercutting the CNT sidewalls (Figure 7) [73]. The patterning of VA-CNTs in large areas was obtained by using laser trimming. The use of a mask allows one to shield part of the VA-CNTs from the intense laser beam while the exposed CNTs are burnt away [74]. Alternatively, the VA-CNTs can be micromachined with the focused pulsed laser beams to produce columns with controlled size and shapes [75].

To conclude this part, the preparation of micropatterned VA-CNTs has been widely studied and significant advances in the methodology have been achieved in order to match the needs for device applications. Two strategies have been developed: the selective growth of VA-CNTs on pre-patterned substrates, and the post-patterning of CNTs films by using laser beams to selectively trench them, leaving the desired pattern behind.

### 3 Functionalization of VA-CNTs

We now focus on the functionalization of vertically aligned carbon nanotube samples. First, we describe the grafting of fluorine-, oxygen- and nitrogen-containing chemical groups at the VA-CNT surface. Next, we review the functionalization of CNTs with nanoparticles such as Cu, Ag, Au, Pt, Pd or TiO_2_. Then, we present polymer-based functionalization and the grafting of biomolecules (DNA molecules, glucose molecules, proteins, etc.) on VA-CNTs for biological applications. Finally, we present some less-common functionalization methods.

#### 3.1 Functional groups

**Fluorination of VA-CNTs:** During the past decade, fluorinated non-aligned carbon nanotubes have been intensively investigated because of their potential applications [76]. Fluorination has been reported to impact on the morphology and on the physical and chemical properties of the CNTs, which can be used, for instance, as new precursors for chemical synthesis due to their better solubility and the creation of weaker C–F bonds [76]. Fluorinated CNTs are better electron acceptors, i.e., more prone to interact than pristine CNTs because both the Fermi energy level and conduction bands shift towards lower energy upon fluorination.

Regarding VA-CNTs, Dickrell et al. showed that the friction coefficient of oriented MWCNTs films depends on the temperature of the sample and the chemical groups at the surface [77]. In 2007, this problematic stirred the curiosity of Ler et al. [78]. Their work revealed the dependence of the friction coefficient on other parameters (CNT functionalization or CNT sidewall morphology, for instance). This value is determined by using a frictional force microscopy (FFM). The principle is the following: an atomic force microscopy (AFM) tip is pushed vertically into the film, between the nanotubes, and the lateral force experienced as the tip moves through the film laterally is measured [79]. Under this condition, it is clear that the CNT sidewall plays a key role in determining the coefficient of friction. Moreover, the CNTs being hydrophobic [80], the water meniscus between the AFM tip and the CNT sidewall can be a hindrance to the tip displacement through the CNT forest and can disturb the measurement. To clarify these points, they accomplished a comparison between friction coefficient measurements for VA-CNTs modified by CF_4_- or O_2_-plasma treatment, under normal room humidity or reduced ambient humidity. The data revealed that the functionalization incorporates chemical species into the VA-CNTs film and modifies the wettability of the sample as well as the CNTs arrangement (according to the gas used in the plasma chamber). Consequently, the value of the coefficient of friction is modified. By contrast, the effect of the humidity on the coefficient of friction was found to be insignificant.

**Figure 8:**
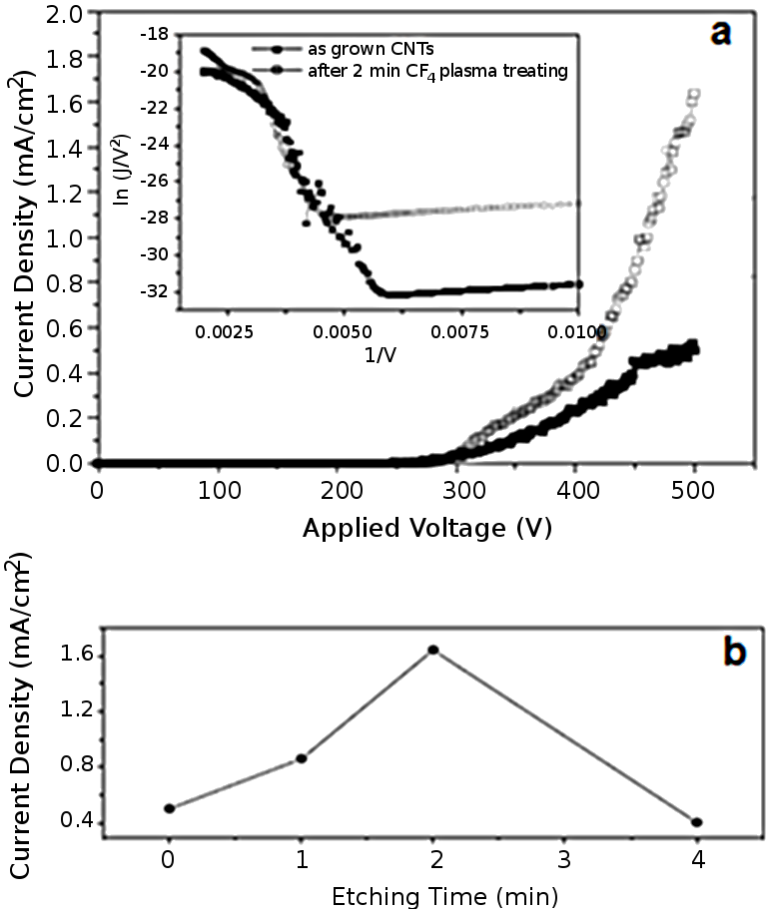
(a) Field-emission data of a CNT film before and after 2 min of CF_4_ plasma treatment. Inset is the corresponding Fowler–Nordheim plots. (b) Dependence of the field-emission current density of aligned multiwall CNTs on the CF_4_ treatment time under an applied voltage of 500 V. Adapted with permission from [88]. Copyright 2005 Elsevier.

Because of their high aspect ratio and high chemical stability, carbon nanotubes can also find practical applications as electron-field emitters in flat-panel displays [81–82]. The major inconvenience is the weak field-emission performance. However, it was demonstrated that a fluorine-based functionalization of carbon nanomaterials such as diamond films [83] or amorphous carbon nanoparticle films [84] increases the yield of the phenomenon. The fluorination of carbon nanofibers [85] and SWCNTs [86] was also underlined. Chung et al. extended it to aligned MWCNTs samples [87]. The proposed solution consists of a layer-by-layer deposition method, involving alternately the deposition of a thin layer of carbon nanotubes and the exposure of its surface to a CF_4_ plasma. The advantage of these combined techniques is the continuous elimination of the unwanted amorphous-carbon and graphite phases forming in the film, resulting in the production of high quality CNTs at low substrate temperatures. Moreover, the CF_4_ treatment opens the ends of the nanotubes, leading to an increase of the emission currents. A similar study was performed by Zhu et al. (Figure 8) [88]. They exposed VA-CNTs films to CF_4_ plasma and evaluated the dependence of the field-emission properties on the exposure time. Results highlighted an optimal exposure time of two minutes, giving the higher field emission current, as well as a modification of the physical and chemical properties of the CNTs due to the plasma process (i.e., fluorination in CF_4_ of the CNTs, defect-density increase and opening of the CNT caps).

**Figure 9:**
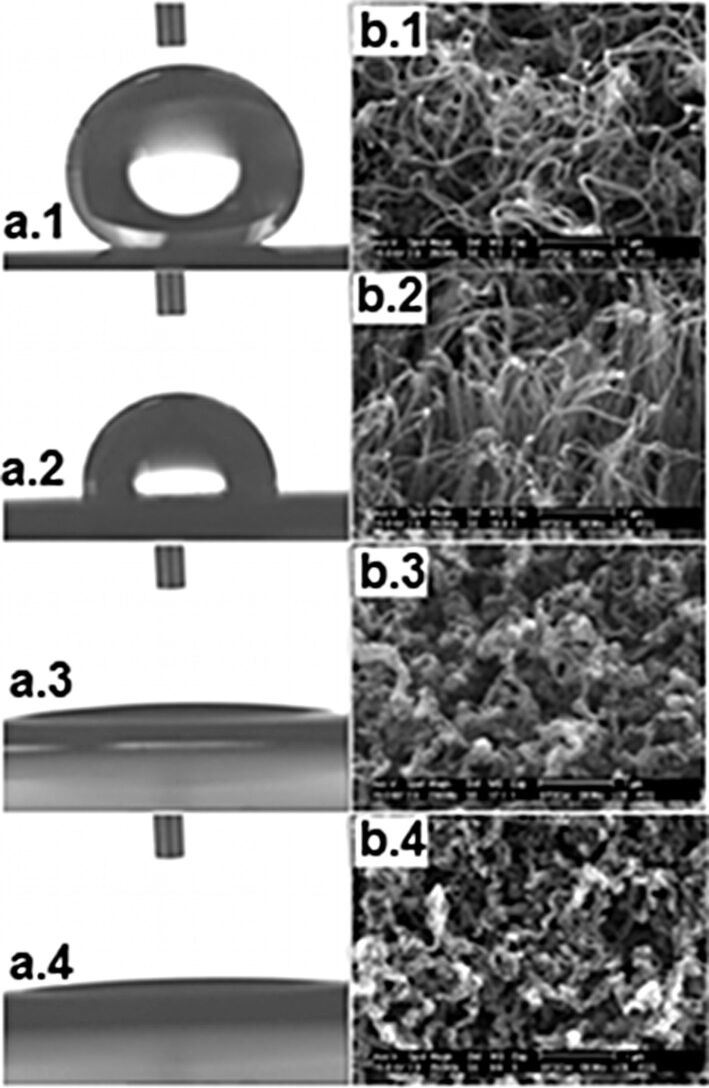
Effect of oxygen plasma functionalization on the VA-CNTs. (1) Optical microscopy images of the contact angle between deionized water (magnification X200) and (2) FEG-SEM images presenting the morphological structure of VA-CNTs. Figure a.1 and b.1 shows the as-grown VA-CNT. Details of oxygen plasma etching: (Figure a.2 and b.2) 1 min., (Figure a.3 and b.3) 2 min. and (Figure a.4 and b.4) 5 min. Adapted with permission from [91]. Copyright 2012 Elsevier.

**Oxidation of VA-CNTs:** As-grown VA-CNTs are superhydrophobic [89]. In 2010, Ramos et al. [90] emphasized that a post-treatment by using oxygen pulsed direct-current (DC) plasma can modify radically the wettability of VA-CNTs. The plasma treatment allows the grafting of oxygen-containing groups onto the VA-CNTs tips, altering the polarity of the sample and leading to a more hydrophilic surface. Ramos et al. showed that a CO_2_ laser irradiation post-treatment can reverse the phenomenon. It totally removes the grafted groups and re-establishes the hydrophobic character of the sample. They reported the ability to control the VA-CNTs wettability (from superhydrophilicity to superhydrophobicity) by combining both techniques. The change in the wettability of the VA-CNTs by grafting oxygen groups was also demonstrated by Lobo et al. [91] (Figure 9). They showed that an oxygen DC plasma etching post-treatment can modify radically the wettability of VA-CNTs films (oxygen flow rate of 1 sccm, at a pressure of 85 mTorr, −700 V, at a repetition rate of 20 kHz). The peculiarity of this treatment is the extremely brief treatment time (1, 2 or 5 min).

Many transport applications (e.g., controlled drug delivery or molecular sensing [92]) require nanoporous membranes with a precise pore diameter, a perfect vertical orientation of the pores and a high uniformity. Until now, their manufacturing represents a complex challenge. Using a VA-CNT film, the inner core diameter of the tubes can be set by the catalytic particle size [93] and the perfect vertical orientation is guaranteed [94–95]. Based on these synthesis characteristics, Majumder et al. [96] reported the engineering of VA-CNTs membranes in four steps: (i) growth of aligned MWCNTs by chemical vapor deposition, (ii) CNT filling with polystyrene, (iii) HF etching in order to separate the composite film from the substrate, and (iv) H_2_O plasma oxidation in order to remove excess surface polymer and Fe nanocrystals at the CNT tips. The final product was a nanoporous membrane with carboxylate functions at the CNT tips. After further chemical functionalization, the nanoporous membrane showed an improvement of the selectivity of the chemical transport across its peculiar architecture. The attachment of oxygen-containing groups on CNTs is often a preliminary step before subsequent functionalizations, since they form active sites where other chemical groups will preferentially graft [97].

We have mentioned the interest in oxygen-containing groups grafting onto VA-CNTs to improve the wettability and the transport properties. Let us focus now on the electrical properties. Kim et al. [98] carried out mild oxygen plasma treatment on SWCNTs aligned between two electrodes. They found that the structural defect density on the sample surface increased linearly with the plasma treatment time. In parallel, they observed an exponential dependence of the resistance. No significant modification of the carrier concentration and tube–tube interaction was noticed. Considering that the electronic transport is driven by the localization mechanism (i.e., a localization of the electron states at the defect sites), Kim et al. explained the correspondence between (i) the linear behavior of the structural defect density with time and (ii) the exponential behavior of the resistance. This observation is important for different electrical devices. Ye et al. [99] electrodeposited molybdenum oxide (MoO_x_) on VA-VNTs forests to develop electrochemical sensors for the detection of bromate. Brown et al. [100] investigated the potential use of VA-CNTs as neural stimulation electrodes. They performed flash oxidation (short exposition time of 20 min) of the VA-CNTs in O_2_ (100 sccm) at various temperatures (between 200 °C and 500 °C) aiming at improving their charge storage properties. Various approaches were tested: (i) purify CNTs by removing carbonaceous impurities, (ii) create active sites for the grafting of hydrophilic oxygenated groups, (iii) attach oxygenated groups on the surface, and (iv) heat the sample. The consequences were an improvement of the electrolyte penetration as well as the wettability, the capacitance, and the charge-storage properties.

Rechargeable lithium-ion (Li-ion) batteries are based on the motion of lithium ions from the negative electrode to the positive electrode when being used, and inversely when charging. Their current limitation comes from their poor performance in terms of energy and power densities, safety and lifetime. Much attention is focused on the electrodes and electrolyte technology. Lu et al. [101] developed vertically aligned carbon nanotubes with and without a coaxial layer of vanadium oxide (V_2_O_5_) as cathode and anode, respectively. Due to their unique properties (e.g., large surface area, electrical conductivity, regular pore structure, electrolyte accessibility, charge transport), they are candidates for replacing traditional electrodes. Instead of traditional organic electrolytes, they used ionic liquids because of their nonflammability, nonvolatility, nontoxicity, large electrochemical window, and wide liquid-phase range. Practically, VA-CNTs were etched by H_2_O plasma in order to open the extremities of the nanotubes prior to an electrochemical deposition of V_2_O_5_ on the sidewalls of the tubes. Opening the tips facilitates the penetration of the electrolyte inside the composite electrode.

**Figure 10:**
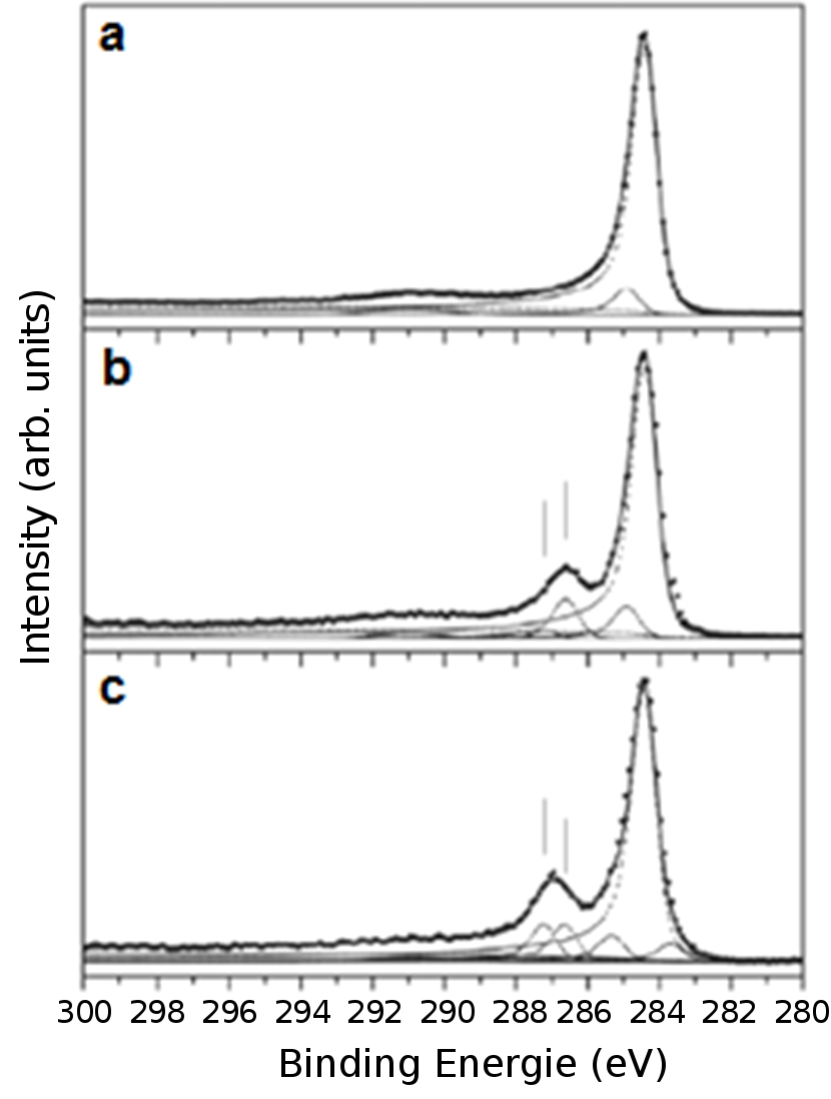
XPS analysis of (a) pristine vertically aligned MWCNTs and oxygen-plasma-treated MWCNTs for (b) 5 min and (c) 30 min. Vertical lines indicate the components associated with the oxygen functionalization. Adapted with permission from [102]. Copyright 2011 American Chemical Society.

**Figure 11:**
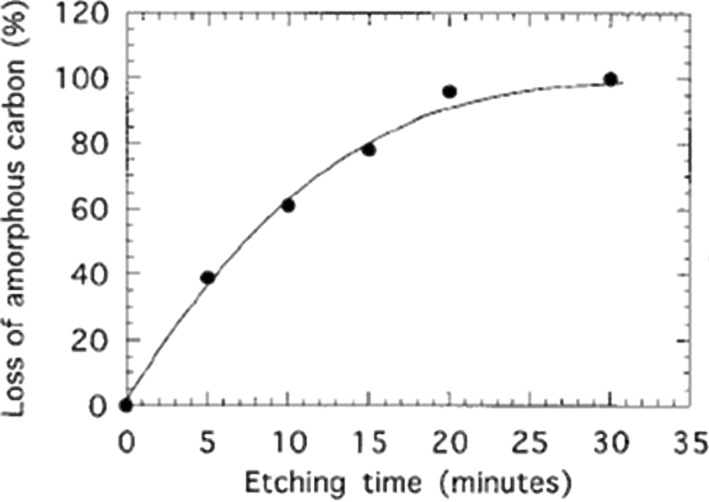
Percentage weight loss of amorphous carbon as a function of the H_2_O-plasma etching time. The H_2_O-plasma etching was performed at 250 kHz, 30 W, and 0.62 Torr. Adapted with permission from [21]. Copyright 2002 American Chemical Society.

Oxygen-based functionalization by plasma techniques can lead to morphological and chemical modifications of the nanomaterials [102–103] (Figure 10). Functionalization of vertically aligned MWCNTs performed by using atomic oxygen, generated by a microwave plasma, was reported to graft oxygen functional groups onto the tips of the VA-MWCNTs, without perturbation of the CNT alignment and structure. The CNT tips are more reactive than the CNT sidewalls [104–105]. In consequence, an opening of the VA-CNTs was observed. Radio-frequency Ar/O_2_ plasma is also able to bring about modifications of the atomic composition and the structure of CNT forests. Zhao et al. [106] performed a detailed experimental investigation of the influence of the plasma parameters (work pressure, gas flow ratio, and plasma power among others) on the surface morphology and the chemical composition of VA-CNTs. As opposed to the wet chemical treatment, the plasma treatment does not destroy the vertical alignment of the nanotubes. By contrast, a significant surface morphology alteration is perceptible: nanotube tips agglomerate together, leading to the appearance of small nanotube bundles. It was proven that the agglomeration is independent of the oxygen concentration in the plasma chamber, but depends strongly on the work pressure and the plasma power. Besides, it was shown that the oxygen-containing compounds are exclusively grafted on the outer surface of the VA-CNT forest. A radio-frequency glow-discharge H_2_O-plasma etching method was used in 2002 by Huang and Dai [21], to purify the VA-CNTs. During VA-CNT synthesis, a thin layer of amorphous carbon covers the aligned nanomaterial film [107] constituting an obstacle for certain applications. Water plasma etching was used to purify the sample and to remove the amorphous parasitic layer. When the plasma conditions are optimal, this method does not cause observable CNT structure or arrangement spoilage. On the contrary, when the conditions become harsh, a selective opening of the top end caps of the VA-CNTs is observed. Similar results were demonstrated both for VA-CNT continuous films and VA-CNT micropatterned films (Figure 11). Recently, water-vapor plasma was likewise used by Hussain et al. [108]. They showed that the plasma treatment (i) introduced defects in the CNT structure, (ii) removed catalyst molecules present at the CNT tips after the CNT synthesis, and (iii) decreased the CNT diameter. Depending on the plasma parameters, it was possible to choose the grafted functional group between carboxyl and hydroxyl, thus tuning the electrochemical properties of the VA-CNTs.

**Figure 12:**
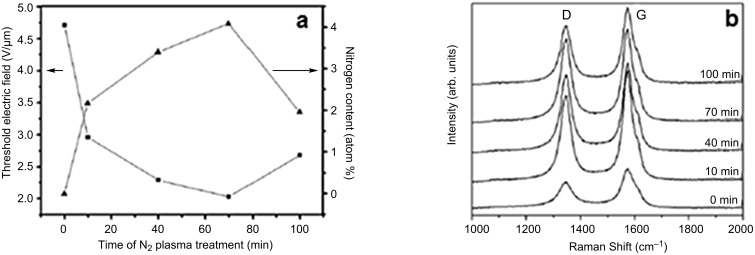
(a) Variation of threshold electric field and nitrogen content with the duration of the nitrogen plasma treatment. (b) Raman spectra of the CNTs after different times of nitrogen plasma treatment. Adapted with permission from [109]. Copyright 2009 Elsevier.

**Nitration of VA-CNTs:** Fluorination of VA-CNTs increases their field-emission performance. Another solution lies in the utilization of post-growth nitrogen plasma treatment. This method was proposed by Lai et al. [109] in 2009. Patterned VA-CNTs, with an ideal hexagon arrangement, were used. Nitrogen doping was incorporated in the CNT bundles by nitrogen RF-plasma treatment (20 W of power, at a pressure of 27 Pa) with various exposure times (10–100 min), indicating the correlation between the duration of the nitrogen plasma treatment, i.e., an optimal doping concentration of impurities and the threshold electric field. (Figure 12) Lai et al. recorded the lowest threshold electric field (with a value of 2.3 V/µm) for the highest nitrogen content of CNTs (4.08 atom %), i.e., for a 70 min nitrogen plasma treatment. The field emission characteristics of CNTs depend on their structural defects [110].

The fuel cell [111], discovered in 1839 by Sir William Grove [112], is a device in which the production of electricity is due to oxidation on a fuel reducer electrode coupled to the reduction of an oxidant on the other electrode. The oxygen reduction reaction plays a key role in the performance of the cell [113–114]. The traditional cell has many drawbacks including high fabrication costs and the spoilage of the platinum electrode with time [115]. VA-CNTs functionalized with electron-accepting nitrogen atoms prove to be a potential candidate to replace traditional Pt-electrodes [116–118].

#### 3.2 Nanoparticles

**Figure 13:**
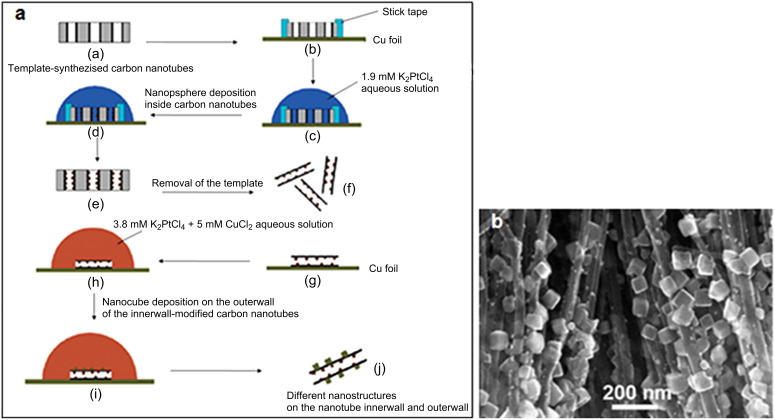
(a) Procedures for the nanotube inner wall modification and the asymmetric modification of the nanotube inner wall with Pt nanospheres and the outer wall with nanocubes. For the sake of clarity, only a few of the many CNTs on the Cu foil are shown. (b) SEM image of aligned CNTs decorated with Pt nanocubes. Adapted with permission from [119]. Copyright 2006 American Chemical Society.

In 2006, Qu et al. [119] developed a method to decorate selectively the walls and the tips of CNTs with metallic nanoparticles (NPs) controlled in size and shape (e.g., Cu, Ag, Au, Pt and Pd nanoparticles). The principle is the following: VA-CNTs are produced by the template-synthesis method by using alumina membrane templates with a pore size of about 200–500 nm; the assembly is then immerged in an aqueous solution, in which the NPs production will occur. Depending on the conditions (metal salt concentration, exposure time), the shape and size of the NPs can be controlled. A subsequent dissolution of the alumina template in aqueous HF retrieves individual nanotubes for further manipulations. For instance, it is possible to obtain an asymmetric functionalization by attaching one kind of NPs on the inner wall of the CNTs and another kind of NPs on the outer wall (Figure 13).

TiO_2_ stirs the curiosity of scientists because of its remarkable semiconducting and photoelectronic properties. Hence, integrated systems combining CNTs and TiO_2_ NPs are potential candidates for photocatalytic or optoelectronic systems, taking advantages of both materials. Yang et al. [120] carried electrophoretic coating of VA-CNTs with TiO_2_ NPs in order to create coaxial nanowires. In parallel, they synthesized aligned TiO_2_ nanotubes and nanomembranes, using the VA-CNT film as a template. All these products have novel photocurrent and photoinduced properties.

**Figure 14:**
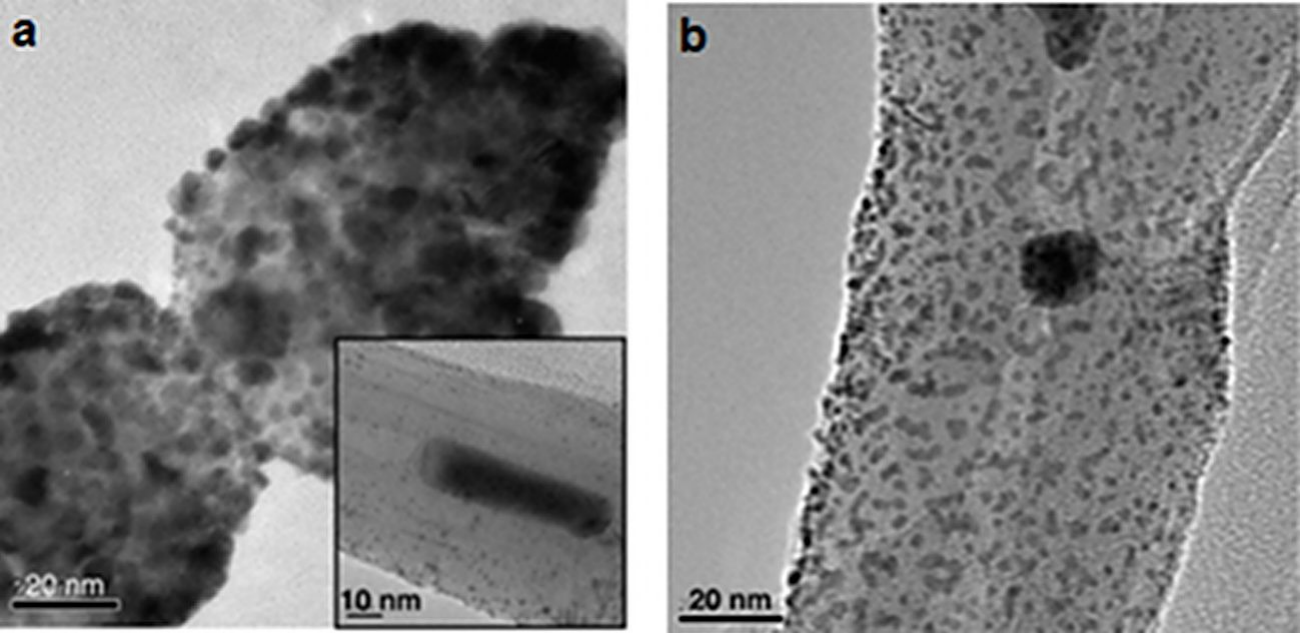
HRTEM images of (a) Pt-NPs-covered CNT tip; inset shows individual particles on the ends of CNTs. (b) individual NPs and clusters along the middle length of the CNT. Adapted with permission from [121]. Copyright 2010 Elsevier.

More recently, the functionalization of VA-CNTs arrays with platinum nanoparticles was examined by Soin et al. [121]. A method combining microwave-plasma-enhanced chemical vapor deposition and DC sputtering was employed in order to synthesize such samples. The alignment of tubes was not perturbed, no physical damage and no etching were observed. Dispersed NPs with diameters close to 2–3 nm were formed at the tip of the tubes. In contrast, at the middle of the tube, NPs with diameters close to 3–5 nm or NPs clusters were observed (Figure 14). The NP spatial distribution depends on the defect density distribution along the length of the tube, since defects are nucleation sites for NP growth [122]. Functionalization of VA-CNT arrays with platinum nanoparticles is promising in fuel-cell development [123]. Similar samples, i.e., platinum decorated VA-CNTs, were produced by Dameron et al. [124] using atomic layer deposition (ALD). First the VA-CNTs were modified by chemical functionalization with a trimethylaluminium (TMA) monolayer or ex-situ Ar, O_2_ or Ar/O_2_ RF-plasma functionalization. Then, platinum was deposited by ALD. The gas-phase functionalization route was preferred in order to control the nucleation sites and to improve the Pt coverage and uniformity on the CNTs. The authors showed that the preliminary treatment, especially the O_2_ plasma treatment, is effective to create nucleation sites and to favor the deep Pt infiltration inside the CNTs array.

#### 3.3 Polymers

In recent decades, many publications have gathered together the tremendous progress made in the understanding of polymer functionalization of VA-CNTs. The success in this field offers a wide range of technological opportunities in a myriad of applications. For instance, in order to enhance the field-emission properties of VA-CNTs, a two-step method can be applied [125]. It consists of sheathing the nanotubes with a thin layer of polymer coating by means of a radio-frequency hexane-plasma treatment, followed by a water-induced restructuring. Another example is the synthesis of conducting coaxial nanowires, useful for optoelectronic and sensing applications. Aligned carbon nanotubes were coated uniformly by electrochemical deposition with an appropriate conducting polymer [126–127].

In order to manufacture plastic solar cells, transparent and flexible conductors are required. Traditionally, indium tin oxide (ITO) is deposited on flexible substrates. The inconvenience is the weak resistance to acid, the weak resistance after repeated strain and the weak conductivity in comparison to glass [128]. Peng [129] developed a CNTs/polymer composite material by spin-coating a polymer solution onto a VA-CNTs sample, followed by the evaporation of the solvent. The product has high optical transparency, robust flexibility and good conductivity.

**Figure 15:**
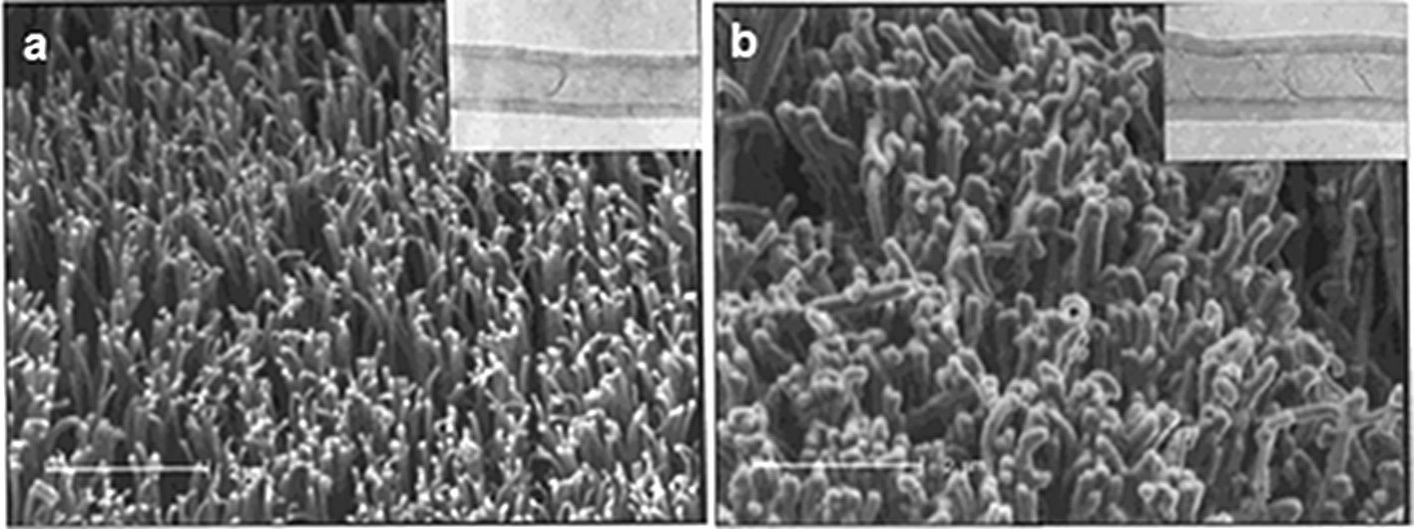
SEM micrographs of the aligned carbon nanotubes (a) before and (b) after plasma polymerization of acetaldehyde. The insets show TEM images of an individual nanotube (a) before and (b) after being coated with a layer of the acetaldehyde-plasma-polymer (optimized conditions for the polymerization of acetaldehyde: 200 kHz, 20 W and a monomer pressure of 0.3 Torr for 5 min). Adapted with permission from [130]. Copyright 2001 American Chemical Society.

Chen et al. [130] used a radio-frequency glow-discharge treatment and chemical reactions post-treatment to graft polysaccharide chains onto a VA-CNT surface. After functionalization, the sample is highly hydrophilic and biocompatible, and presents little perturbation of the CNT structure. This highly hydrated coating offers possibilities to use VA-CNTs in biological systems (Figure 15).

**Figure 16:**
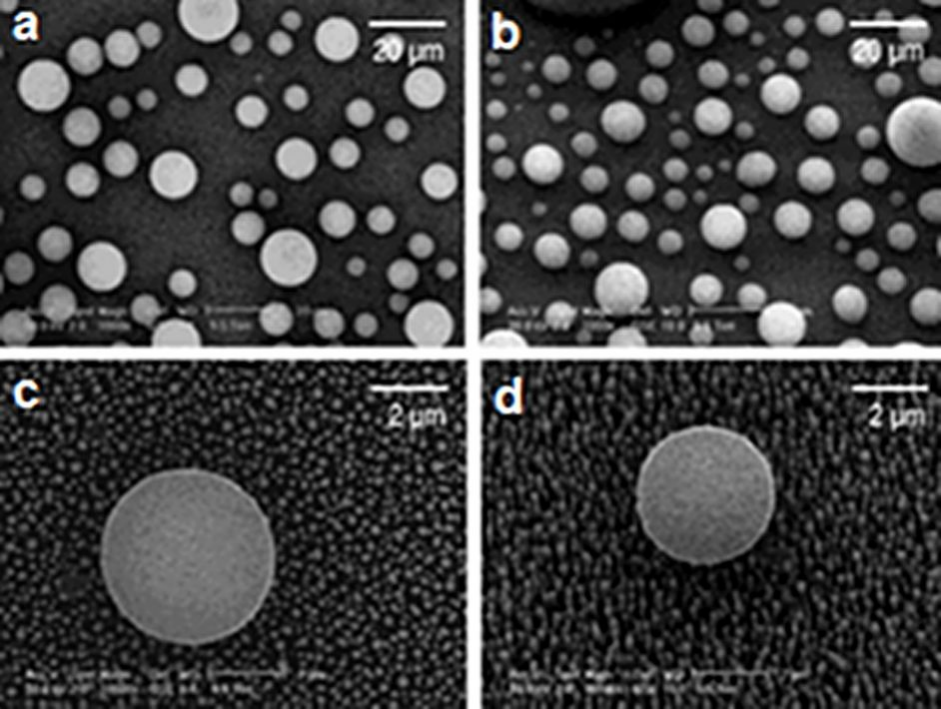
SEM images of water droplets on CNT films. (a) Top-down view of micron-sized water droplets suspended on the PTFE-coated films, (b) 15° tilt view at the same magnification, (c) top-down view of a single suspended water droplet in which the PTFE-coated nanotubes are also visible, and (d) 15° tilt view at the same magnification. Adapted with permission from [131]. Copyright 2003 American Chemical Society.

The wettability properties of VA-CNTs can be changed by grafting oxygen functional groups, but also by polymer functionalization. Lau et al. [131] favored a bio-inspired approach to the problem and mimicked designs found in nature. In certain plants such as the lotus leaf, water droplets roll on the surface and remove dust particles; this is a self-cleaning behavior and is called the Lotus effect [132]. The origin is the peculiar roughness and the intrinsic hydrophobic behavior of the surface. Based on this observation, the authors enhanced the superhydrophobic effect on CNTs by combining two elements: the coating of VA-CNTs with hydrophobic poly(tetrafluoroethylene) (PTFE) and the nanoscale roughness inherent to the sample (Figure 16).

**Figure 17:**
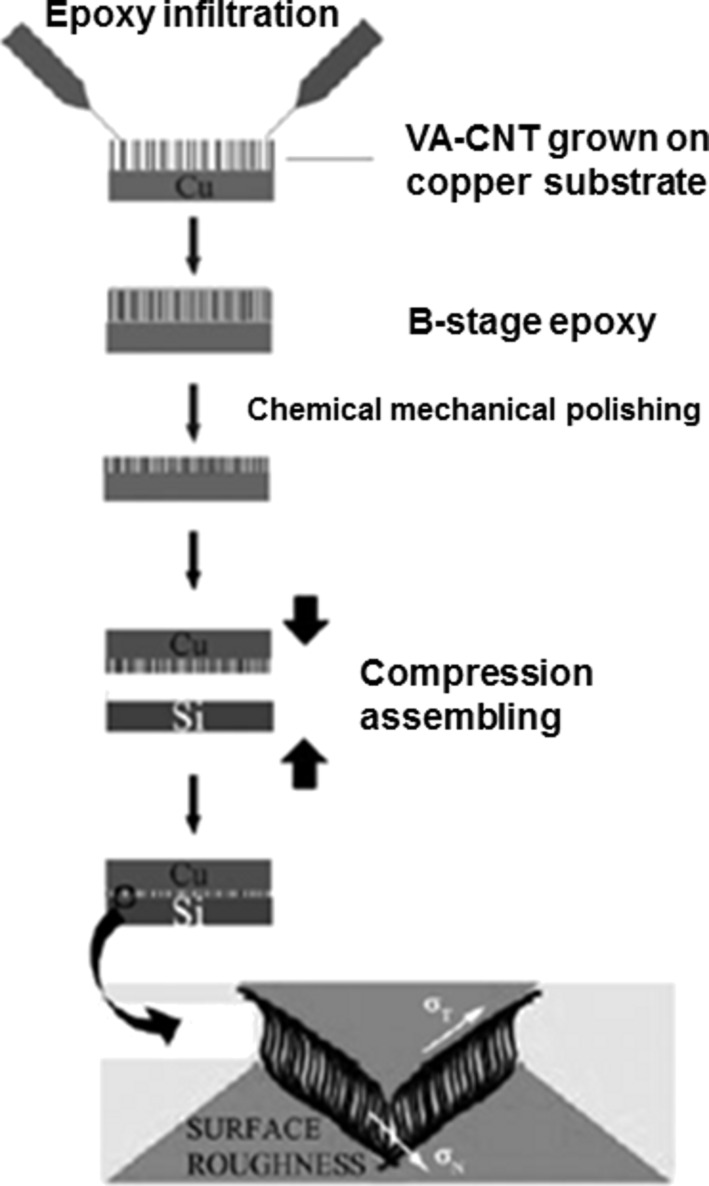
Illustration of the ACNT-epoxy TIM assembly process and the CNT statues at the interface. Adapted with permission from [135]. Copyright 2009 John Wiley and Sons.

The enhancement of the thermal conductivity of a composite enclosure in the direction of its thickness is another illustration of the application of VA-CNT polymer functionalization. Sihn et al. [133] embedded VA-MWCNTs in an adhesive medium (epoxy infused between CNTs) and encapsulated the whole between two adherent media (graphite facesheets). A transition zone made of metallic coatings was inserted between the CNTs tips and the surroundings, after their suitable functionalization. The authors evaluated experimentally and numerically the through-thickness thermal conductivity of the composite sample. They reported that the key components influencing the thermal conductivity are, on the one hand, the thermal conductivity and the size of the metallic transition zone and, on the other hand, the use of highly conductive vertically aligned nanotubes. A preliminary study performed by Huang et al. [134] has laid the foundations for this observation. Lin et al. [135] went a step further by adapting VA-CNTs–epoxy nanocomposites to produce thermal interface materials (TIMs) (Figure 17). A process combining in situ functionalization of VA-CNTs and microwave curing was employed. This device has an ultrasmall coefficient of thermal expansion (CTE), good mechanical load transfer, and good phonon transport across the interface.

The control of the CNT orientation within the polymer matrix (PM) and the control of the interaction between both components are of paramount importance [136]. VA-CNT films are good candidates matching these requirements. In 2002, Nguyen et al. [137] created CNT–PM samples in two steps: first, a nucleic acid grafting at the CNT tips, leading to the opening of their end caps, and then a spin-on-glass deposition inside the VA-CNT array. Hinds et al. [138] and then Chopra et al. [139] used, instead of a spin-on-glass matrix, a polystyrene (PS) matrix. By means of refined experimental examinations, the first group proved an efficient molecular transport through the CNTs cores, opening perspectives in chemical separation and sensing. The second group showed that encapsulating CNTs in a polystyrene matrix protects their sidewalls against oxidation and favors selective and independent functionalization of each end of the CNTs. Moreover, individual CNTs with a chemical group attached at each end can be retrieved by the dissolution of the PS membrane.

**Figure 18:**
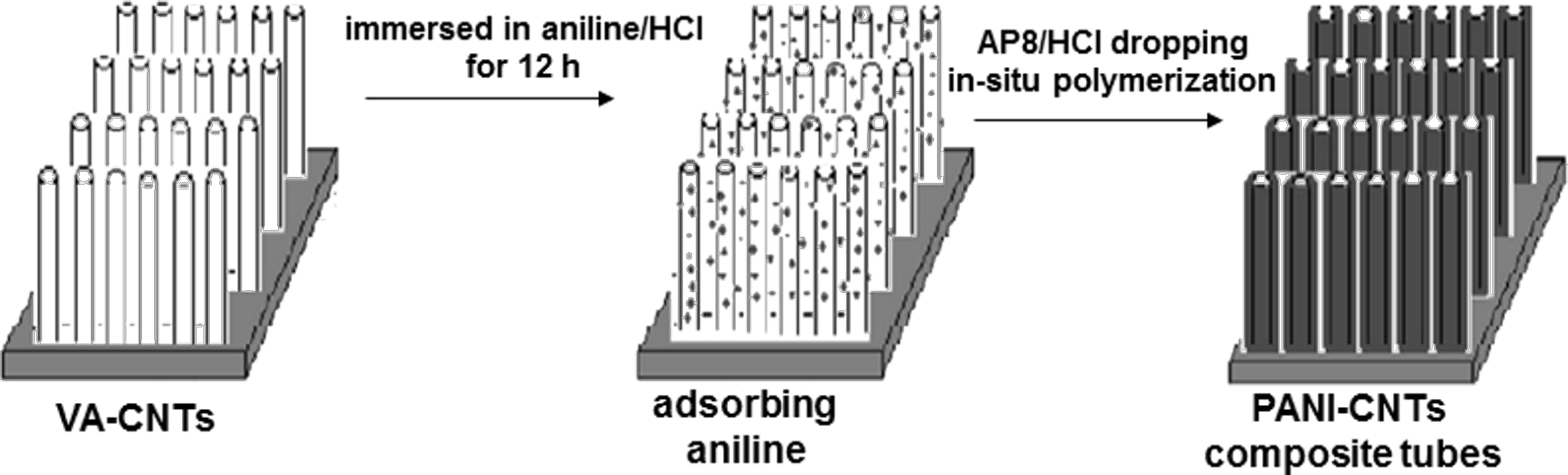
Preparation procedure for organizing PANI/MWCNT nanocomposite-tube films. (a) Aligned MWCNT film grown on quartz substrate by catalytic pyrolysis. (b) The aligned MWCNT film immersed into aniline/HCl solution at 0 °C for 12 h. (c) Polymerization takes place on the MWCNT surface and forms an ordered PANI/MWCNT nanocomposite-tube film. Adapted with permission from [140]. Copyright 2003 Elsevier.

Feng et al. [140] opted for a polyaniline (PANI) matrix. This material is one of the most conducting polymers (Figure 18). Synthesis and characterization of conducting polymer polyaniline nanofibers was reported by Huang [141]. The major issue related to great disorder of the functionalized fibers was solved by Feng et al. who made well-aligned MWCNTs/PANI hybrid materials. The methodology is the following: (i) VA-CNTs are grown on a quartz substrate by catalytic pyrolysis, (ii) the film is immersed in an aniline/HCl solution (0 °C, 12 h), (iii) polymerization on the CNTs surfaces. The products showed a high-quality structural arrangement and an enhanced electrical conductivity. Many physical properties such as morphology, thermal stability, conductivity and charge carrier mobility are disrupted by this treatment. This can be crucial for applications in the photovoltaic field. We can also mention the work of Raravikar et al. [142] who embedded VA-CNTs into a poly(methyl methacrylate) (PMMA) matrix with a two-step strategy. The first step is the fabrication of a VA-CNTs array followed by a MMA monomer infiltration while the subsequent step is in situ polymerization. Finally, we mention the work of Jung et al. [143] who created flexible CNT–PM samples with soft poly(dimethysiloxane) as the polymer compound. Their product respects the CNTs alignment, is highly flexible and retains its conducting properties even under hard tensile and compressive forces.

**Figure 19:**
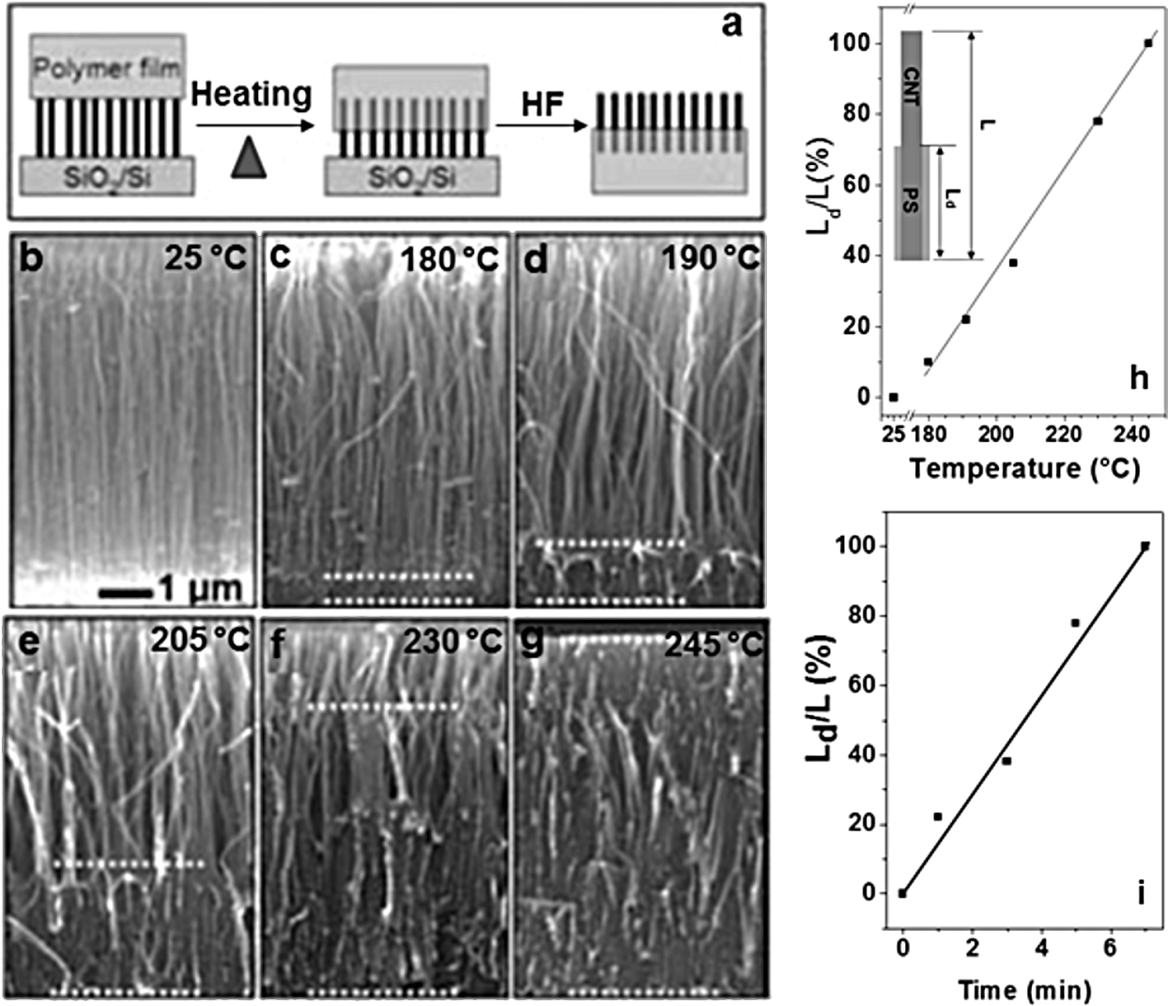
(a) Schematic representation of the VA-CNTs embedded into a polymer matrix by thermal infiltration of the melted polymer into the nanotube forest; (b)–(g) SEM images of (b) the pristine VA-CNT array and (c)–(g) the VA-CNT array after being embedded into PS films by heating at different temperatures for 1 min. The gaps between the dashed lines crossing the polymer coated regions show the approximate embedment length for each of the PS-embedded VA-CNTs; (h) and (i) temperature and time dependence of the embedment length for VA-CNTs embedded into the PS matrix (*L*: nanotube length (≈6 μm), *L*_d_: embedment length, which was estimated from the distance between the two dashed lines in each of the images shown in (c)–(g). Adapted with permission from [144]. Copyright 2007 Royal Society of Chemistry.

As shown in the literature, a large variety of polymer matrices can be used. The principal approach to achieve this CNT–PM mixing is the solution-coating. However, in all these works, the inconvenience was the difficulty in controlling the nanotube length being embedded within the matrix. In 2007, Qu and Dai [144] brought a simple answer: a thin polymer layer is deposited on the surface of a VA-CNT array, followed by heating beyond the melting temperature of the polymer, causing its infiltration inside the array. According to the heating temperature and the exposure time, the resulting composite shows a more or less large polymer-free region (Figure 19). Mechanical insertion of a VA-CNT forest in a spin-cast PM allows the best control of the penetration depth [145]. A post-treatment consisting of the attachment of nanoparticles in the polymer-free region can take place (as discussed in the previous section), the functionalization length being set by the experimental conditions of the synthesis of the VA-CNT–PM composite. Moreover, using the polymer-masking technique twice, it is possible to achieve an asymmetric functionalization of the CNT sidewalls [146]. Finally, using compounds such as iron adds new magnetic properties to the CNT tips.

The polymer-masking methodology was also used to manufacture highly efficient gas sensors [147]. VA-CNTs were embedded in a polymer matrix (e.g., poly(vinyl acetate) or polyisoprene) and two gold electrodes were sputtered onto the surface of the sample. A change of the charge transfer or the capacitance will then indicate the presence of gases in the environment [148–150]. In this context, we mention also the work of Chen et al. [151]. They integrated temperature-responsive polymers in an array of VA-CNTs to produce composite systems with self-cleaning capabilities and/or with abilities to controllably release objects trapped within the systems. For example, we can imagine the production of antifouling devices, gecko-like artificial devices, functional membranes, or sensors.

Wardle et al. [152] assessed the density of VA-CNTs that it is possible to insert in a PM with a two-step technique (a mechanical densification of VA-CNT forests followed by a capillarity-driven wetting along the axis of the CNTs). They revealed that the theoretical limit can be approached, i.e., a distance between two neighboring nanotubes close to the typical length of the polymer chains. The morphological features of the sample (CNT alignment and distribution, polymer morphology) seem to be conserved despite this close packing.

Based on the interaction between carbon nanotubes and polymer or ceramic (e.g., silicon nitride), membranes including VA-CNTs can be fabricated with potential applications in novel ultrafiltration and sized-based exclusion separation devices. These membranes present many advantages in comparison with traditional membranes. Indeed, studies highlighted an enhancement of the properties of the flow of liquids or gases through the CNTs cores [153]. In the case of CNTs being used as transport channels, we draw the attention of the reader to the fact that the VA-CNTs must be opened (by example by plasma oxidation) and that this treatment introduces functional groups modifying the transport properties [154].

**Figure 20:**
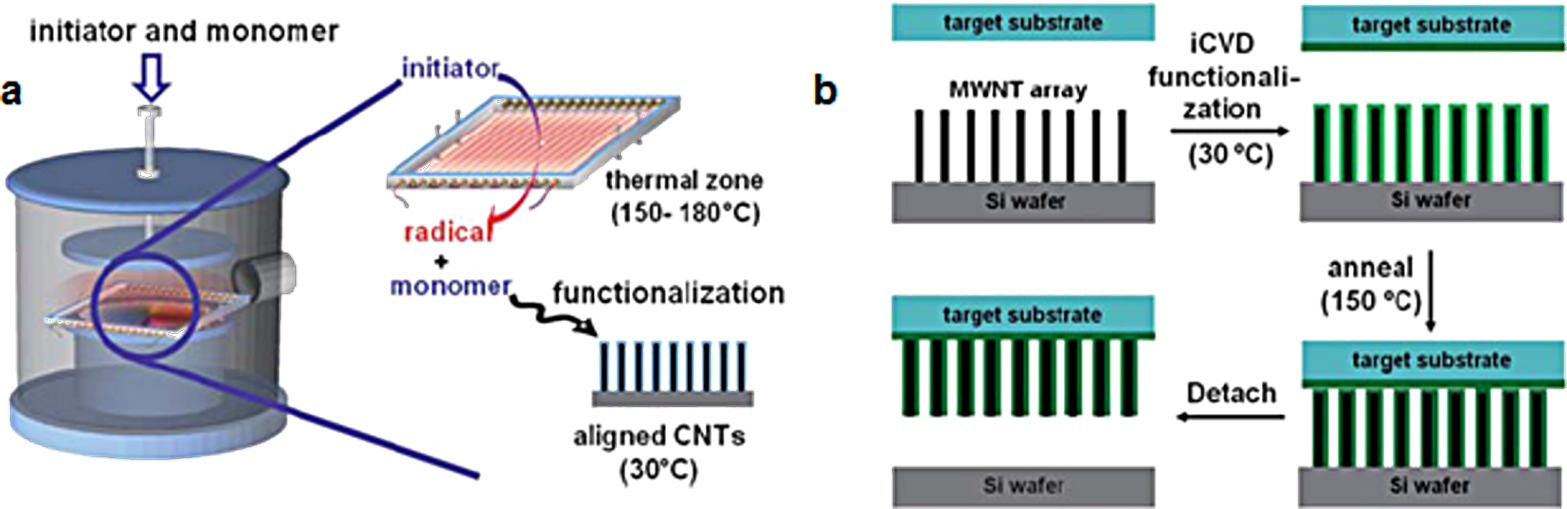
(a) Schematic illustration of the iCVD functionalization process of aligned CNTs and (b) schematic of the MWCNT transfer process. Adapted with permission from [156]. Copyright 2011 Royal Society of Chemistry.

In this section, various techniques allowing functionalization of VA-CNTs were presented, such as the polymer-impregnation technique, polymer-masking technique, sheathing technique and in situ polymerization. The vapor-based technique, a solventless method, was also presented. In 2005, He et al. [155] deposited uniformly thin carbon fluorine films on the surface of aligned carbon nanotubes by means of a plasma-polymerization treatment. In 2011, Ye et al. [156] applied initiated the chemical vapor deposition (iCVD) method, a one-step polymer deposition method without any liquid medium (Figure 20). Initiator molecules thermally break down into radicals at relatively low temperature. Then, a radical polymerization of the specific monomers begins at the surface of the substrate. In their work, Ye et al. used an array of VA-CNTs on a silicon wafer, a *tert*-butyl peroxide (TBP) initiator, and a glycidyl methacrylate (GMA) monomer. After iCVD, the CNTs alignment is preserved. Furthermore, the sample porosity and the deposition uniformity along the CNTs sidewalls can be controlled with the exposure time and deposition rate. The restrictions to the VA-CNT growth are the limited choice of substrates and the poor adhesion between both elements. Hence, Ye et al. intended to transfer the functionalized VA-CNT array to another substrate. They performed similar polymerization treatment on the novel substrate, placed the functionalized VA-CNTs array in contact with the novel substrate, and annealed the system under vacuum at 150 °C. The result was the separation of the CNTs array from the silicon substrate and the attachment to the novel substrate. This low temperature flip-over transfer method preserves the CNTs alignment, gives a strong adhesion between the CNTs and the novel substrate, improves the mechanical properties of the system, and enhances the stability towards wetting (Figure 20).

Recent work on the covalent functionalization of VA-CNTs with polystyrene was published by MacDonald et al. [157]. The originality lies in the type of samples they used: instead of using CVD aligned nanotubes, a method reported by Yu et al. [158] was preferred. In this case, VA-CNTs were attached to a silicon(100) substrate by chemical anchoring directly to the surface. The silicon surface was hydroxylated while the surface of the nanotubes was acid treated and functionalized with carboxylic moieties. MacDonald et al. succeeded in modifying the sample in situ. The carboxylic group was derivatized to bis(dithioesther) moieties, which act as a chain transfer agent (CTA), before application of a reversible addition fragmentation chain transfer (RAFT) polymerization of polystyrene on the surface. The results of this work are important for the design of water-treatment membranes, solar cells, or biochemical sensors.

#### 3.4 Biomolecules

**Figure 21:**
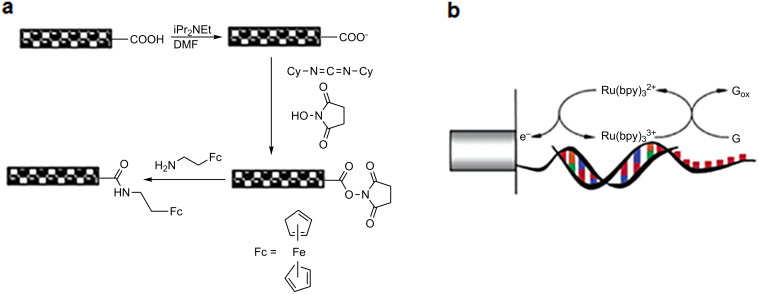
(a) The functionalization process of the amine-terminated ferrocene derivative to CNT ends by carbodiimide chemistry and (b) the schematic mechanism of Ru(bpy)_3_^3+^-mediated guanine oxidation. Adapted with permission from [162]. Copyright 2003 American Chemical Society.

A major issue is the fast and reliable detection of minute quantities of enzymes, proteins or DNA molecules for diagnostic purposes. For this reason, devices with an easy measurement method, high sensitivity, high selectivity and low production cost are required. It has been proven that CNT arrays are a good candidate. Dwyer et al. [159] as well as Willams et al. [160] grafted modified DNA strands at the open ends and defect sites of SWCNTs. This was an initial step to the fabrication of self-assembled molecular-scale electronic systems. Thereafter, researchers have moved away from CNT powder and have focused on VA-CNTs. Indeed, VA-CNTs present many advantages such as a large surface area in order to offer a maximized contact with the analyzed sample or ease of device manufacturing. In 2002, Li et al. fabricated an aligned MWCNT array embedded in a SiO_2_ matrix [161] and functionalized CNT tips with primary amine-terminated oligonucleotide probes [162]. An electrochemical platform, combining such a CNT nanoelectrode array with Ru(bpy)_3_^2+^-mediated guanine oxidation (Figure 21), can detect the hybridization of extremely small amounts of DNA targets (less than a few attomoles).

**Figure 22:**
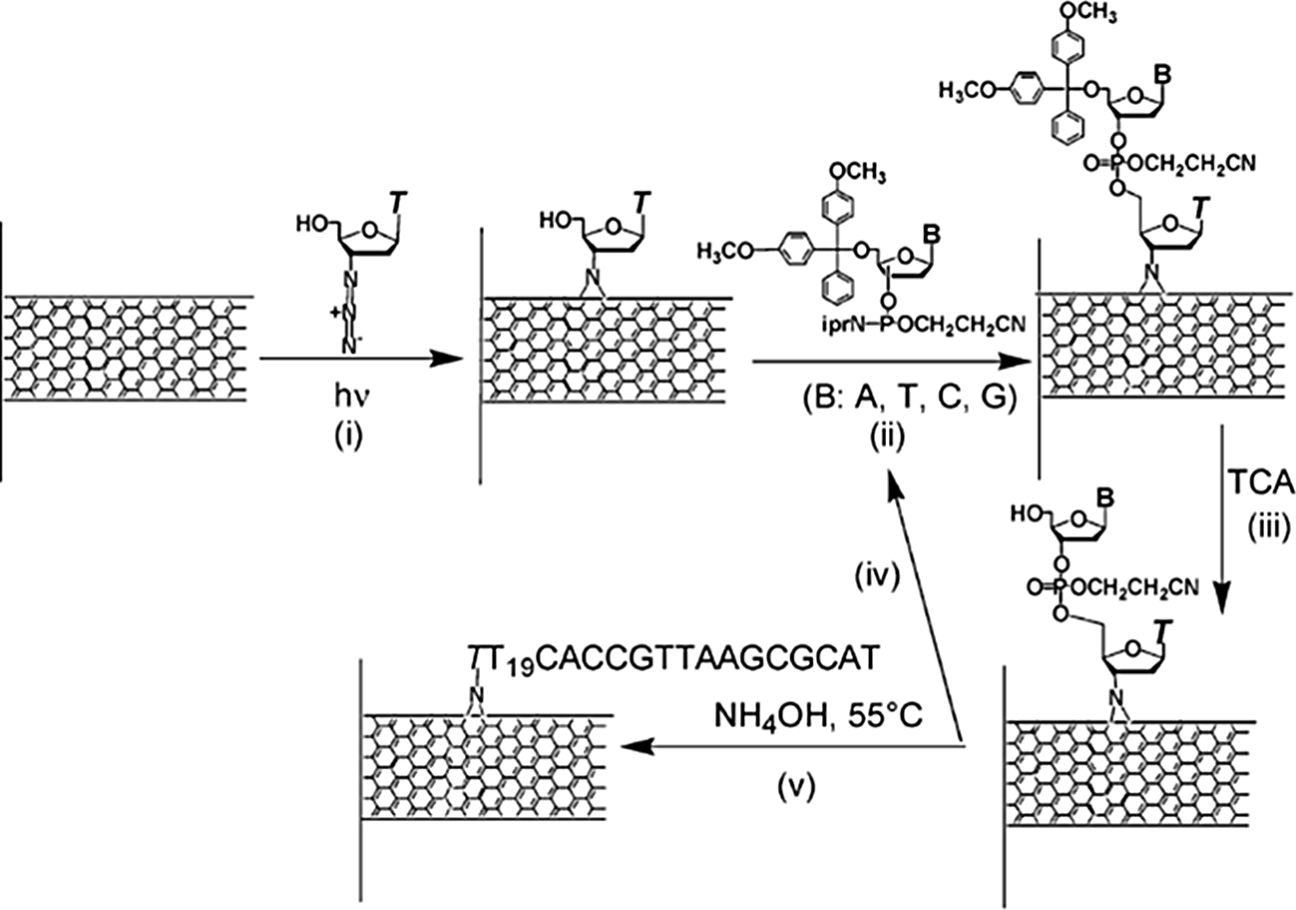
In-situ DNA synthesis from sidewalls of carbon nanotubes photoetched with azidothymidine. Aligned MWCNTs on a solid support are coated with a solution of azidothymidine and are UV irradiated to produce photoadducts, each with a hydroxyl group (i). The hydroxyl group reacts with a phosphoramidite mononucleotide to initiate synthesis of the DNA molecule (ii). Trichloroacetic acid deprotects the hydroxyl group (iii) for reaction with the next nucleotide (iv) and the cycle is repeated until the molecule with the desired base-sequence is made. Finally, the supported nanotubes are heated in ammonia solution to remove blocking groups from the nucleotides (v) to produce DNA-coated nanotubes. Adapted with permission from [164]. Copyright 2004 American Chemical Society.

Gooding et al. [163] showed that a platform composed of VA-SWCNTs and redox protein grafted to the CNTs tips is a good electrical communication channel between the underlying electrode and the proteins. This work represents a major step towards bioelectrical devices. In all these works, only the tip of the nanotubes could be modified. Moghaddam et al. [164], firstly, and He and Dai [165], secondly, proposed a method to attach single-strand DNA chains both to the sidewall and to the tips of the vertically aligned carbon nanotubes. These devices offer novel perspectives in the biosensors domain since they have a high sensitivity and selectivity for probing complementary DNA chains of specific base sequences (Figure 22). In this context, Lin et al. [166] and Pandey et al. [167] fabricated glucose biosensors based on VA-MWCNTs functionalized by glucose oxidase (GOx) molecules. They showed encouraging results in terms of the minimum limit of detection and sensitivity, as well as the possibility for device reuse (up to six months) when stored in a proper environment.

**Figure 23:**
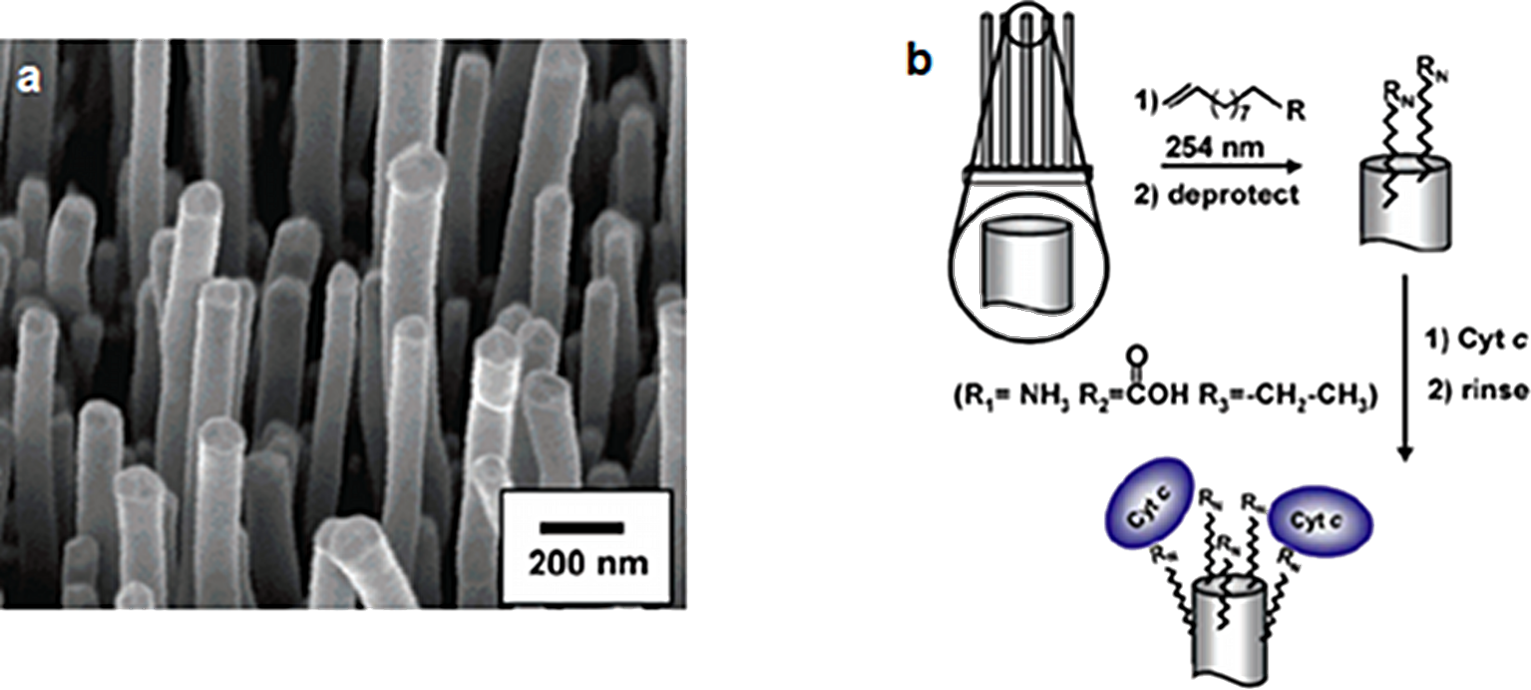
(a) SEM image (taken at a 25° tilt) of a VA-CNFs substrate. (b) Schematic of the functionalization of VA-CNFs with the redox-active protein Cytochrome *c*. Adapted with permission from [169]. Copyright 2006 American Chemical Society.

With a high chemical stability, a large surface area easily accessible to molecules in solution, a quasi-perfect vertical arrangement, a nanoscale size, and a facility to synthesize patterned samples, VA-CNTs are ideal for biological detection. Additionally, their high mechanical strength and their small diameter suggest that VA-CNTs are ideal for intracellular detection [168]. Baker et al. [169] manufactured vertically aligned carbon nanofibers (VA-CNFs)-based electrodes as a platform for biological detection. Using a photochemical functionalization, carboxylic acid functions are grafted on the carbon nanomaterial. Thereafter, these functions act as a preferential site where Cytochrome *c* metalloproteins (small proteins found associated with the inner membrane of the mitochindrion) will be immobilized (Figure 23).

#### 3.5 Other functionalization groups

In the context of the investigation of complex electrocatalytic reactions, Landis et al. [170–171] functionalized VA-CNTs with redox-active molecules (e.g., ferrocene) and studied the electron-transfer properties of the system. They explained that the edge-plane graphite sites along the CNT sidewalls provide anchorage points for the ferrocene molecules. An exclusive study of the role of these sites on the electrochemical properties of carbon nanotubes was also performed [172].

Finally, the properties of VA-CNTs can be changed by doping during the nanotube growth itself. The doping of aligned CNTs has been mostly done with nitrogen [173–174] in order to, for example, enhance the field-emission properties [175], but other elements such as boron have also been studied [176]. The strategy is to use a dopant source with the hydrocarbon compounds during the nanotube growth in order to introduce dopant atoms into the carbon networks composing the nanotube walls. Besides, doping during the nanotube growth itself also activates the nanotube sidewalls and increases their reactivity towards further functionalization. Indeed, a correlation between the crystallinity of nanotube walls and the doping content has been highlighted: the increase of doping content allows a weak crystallinity and a higher defect density (this effect is generally well illustrated by Raman spectroscopy where the D-band (1315 cm^−1^) related to the defects increases with the doping content) [174]. This high density of defects increases the reactivity of the CNTs towards further functionalization.

#### 3.6 Functionalization and CNTs bundling

As mentioned in this review, the functionalization of VA-CNTs has many consequences, actively sought or otherwise. For instance, the tendency that VA-CNTs have to clump together into bundles could be desirable, as in the case of superhydrophobicity/hydrophilicity studies, but in general, it represents a handicap. Indeed, CNT bundling significantly reduces access to the CNT sidewalls and inhibits an effective and uniform functionalization of the tubes. It seems important to include in this review a discussion dedicated to this topic.

**Figure 24:**
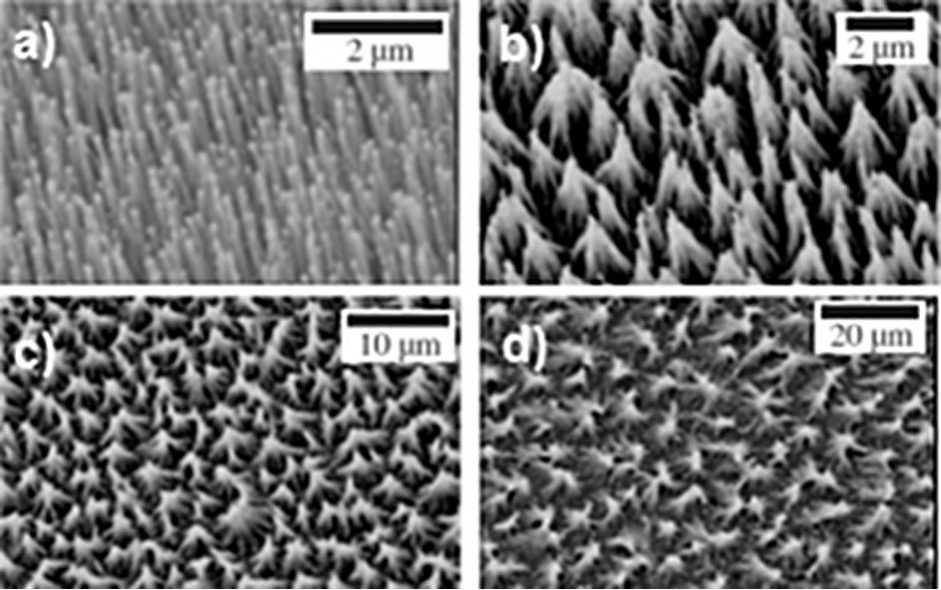
SEM images of superhydrophobic CNT forests, after functionalization with thiols: (a) in the gas phase; (b-d) in ethanol, for increasing initial nanotube length, resulting in characteristic lateral roughness length scale *L*: 1.7, 3.5 and 6 μm (pictures b, c and d). Adapted with permission from [178]. Copyright 2006 American Physical Society.

**CNTs bundling:** In 2004, Bico et al. [177] investigated this bundling topic using an original approach: they dunked a brush of parallel elastic lamellae in a perfectly wetting liquid and, after withdrawal of the brush, they observed the formation of hierarchical bundling patterns. They noted that the lamellae aggregation depended on a balance between the lamellae elasticity and the capillary forces. This coalescence process can take place in the VA-CNT films and influence their structures. This point has been discussed by Joseph et al. [178]. Their concern was the understanding of the influence of the roughness of superhydrophobic surfaces on their hydrodynamic characteristics. Toward this goal, they studied superhydrophobic VA-CNT forests coated by a thin gold layer, functionalized with thiol molecules in gas or liquid phase and included into the microchannels. They reported that the gas-phase mode does not affect the CNTs structure while, in contrast, the liquid-phase mode leads to the bundling of the CNTs, modifying thereby the surface roughness (Figure 24). The bundling was associated to the capillary adhesion occurring during the evaporative drying of the functionalization solvent (ethanol in this case) in the liquid-phase mode. We can also mention the work of Journet et al. [179]. After Lau et al. in 2003 [131], the superhydrophobicity effect on VA-CNTs forests has also gained their attention*.* They functionalized such surfaces with the same route as Joseph et al., i.e., through the anchorage of thiol molecules (in the liquid or vapor phase) on a thin gold coating. They inspected the effect of pressure on the contact angle of a water drop on these superhydrophobic surfaces and showed that they are resistant to high excess pressure (>10 kPa), while keeping their low adhesion characteristics.

**Dry gas-phase functionalization:** To avoid VA-CNT bundling and obtain a very homogeneous surface functionalization, gas-phase functionalization represents an original and alternative route. In this context, in a recent patent [180], Bismarck et al. proposed a one-step process that has the peculiarity of being carried out in situ, in the growth chamber. The principle is the following [181]: CNTs are submitted to a high-temperature treatment (at 1000 °C for 2 h), before being slowly cooled to room temperature; the thermally activated sample is then exposed to a functional organic reactant in vapor form. With this process, the nanotubes have not been wetted and, hence, do not bundle together. In consequence, the surface functionalization is very homogeneous. Another example of in situ gas-phase functionalization is reported by Lin et al. [182]. The VA-CNT synthesis by chemical vapor deposition (CVD) has the inconvenience of a high growth temperature and few substrate materials available. The solution they proposed consists of the separation of the growth process and the CNT assembly. This new transfer technology is based on the functionalization of VA-CNTs. In comparison to the wet chemical process, this technology preserves the alignment of CNTs, introduces few defects to the CNTs, and allows patterning, precise length control, and quality control of the samples. The first step is the in situ functionalization of VA-CNTs during their CVD growth. A small amount of Ar is bubbled through H_2_O_2_ into the furnace chamber during the CVD process. The consequence is the grafting of oxygen-containing compounds onto the sample surface. The second step is the transfer of the functionalized VA-CNT sample onto a novel substrate (metal, polymer or semiconductor substrate) coated by self-assembled monolayers of conjugated thiol molecules on gold surfaces. This transfer technology, characterized by a self-assembled monolayer of conjugated thiol molecules at the interface between the VA-CNTs and the gold surface, is useful for providing both a bonding ligand and the pathway for tunneling or electron transport, and also for phonon transport. Finally, we can also mention the work of Shulga et al. [183]. In 2011, they reported a simple technique to functionalize millimeter-high, aligned carbon nanotubes using nitric acid (HNO_3_) vapor at low temperature, leading to the formation of carbonyl and carboxyl groups on the CNTs. According to the process parameters (temperature, exposure time), one can obtain highly uniform functionalization along the CNT sidewalls, preserve the CNT alignment and minimize the CNT destruction.

## Conclusion

We have reviewed recent developments on the functionalization of vertically aligned carbon nanotubes. This topic is at the forefront of current scientific research, as demonstrated by the wealth of articles in the literature. Depending on the intended application and required properties, different functionalization of VA-CNT films is possible: uniformly over the nanotubes in their entirety, or selectively for the CNT tips and the CNT sidewalls. Two kinds of functionalization coexist: chemical functionalization and dry chemical functionalization, each method having its advantages and its inconveniences. A large variety of potential applications could be developed in a multitude of fields including electron field emitters, chemical and biological sensors, solar cells, optoelectronic devices, selective nanoporous membranes, self-cleaning films, materials with adaptable-wettability, fuel cells, etc.
